# In silico evolution of *Aspergillus niger* organic acid production suggests strategies for switching acid output

**DOI:** 10.1186/s13068-020-01678-z

**Published:** 2020-02-24

**Authors:** Daniel J. Upton, Simon J. McQueen-Mason, A. Jamie Wood

**Affiliations:** 1 Department of Biology, University of York, Wentworth Way, York, YO10 5DD UK; 2 Department of Mathematics, University of York, Heslington, York, YO10 5DD UK

**Keywords:** *Aspergillus niger*, Genetic algorithm, Citric acid, Succinic acid, Evolution, FBA

## Abstract

**Background:**

The fungus *Aspergillus nige*r is an important industrial organism for citric acid fermentation; one of the most efficient biotechnological processes. Previously we introduced a dynamic model that captures this process in the industrially relevant batch fermentation setting, providing a more accurate predictive platform to guide targeted engineering. In this article we exploit this dynamic modelling framework, coupled with a robust genetic algorithm for the in silico evolution of *A. niger* organic acid production, to provide solutions to complex evolutionary goals involving a multiplicity of targets and beyond the reach of simple Boolean gene deletions. We base this work on the latest metabolic models of the parent citric acid producing strain ATCC1015 dedicated to organic acid production with the required exhaustive genomic coverage needed to perform exploratory in silico evolution.

**Results:**

With the use of our informed evolutionary framework, we demonstrate targeted changes that induce a complete switch of acid output from citric to numerous different commercially valuable target organic acids including succinic acid. We highlight the key changes in flux patterns that occur in each case, suggesting potentially valuable targets for engineering. We also show that optimum acid productivity is achieved through a balance of organic acid and biomass production, requiring finely tuned flux constraints that give a growth rate optimal for productivity.

**Conclusions:**

This study shows how a genome-scale metabolic model can be integrated with dynamic modelling and metaheuristic algorithms to provide solutions to complex metabolic engineering goals of industrial importance. This framework for in silico guided engineering, based on the dynamic batch growth relevant to industrial processes, offers considerable potential for future endeavours focused on the engineering of organisms to produce valuable products.

## Background

The natural ability of *Aspergillus niger* to secrete organic acids and enzymes has led to it becoming an established industrial organism, and is currently the chief global source of citric acid. The species belongs to the group known as the *Aspergilli,* which encompasses over 300 species, including the pathogenic *A. fumigatus*, the feed-contaminating *A. flavus*, the model organism *A. nidulans*, as well as *A. oryzae* and *A. niger*, both used industrially. In recent years, the *Aspergilli* have been subject to major advances in systems biology approaches [1], that lay promise for a replacement of traditional strain development methods with more efficient, in silico guided ones. Many decades of random mutagenesis were needed to create the *A. niger* strains used in industry today. Although these industrial strains are already optimised for citric acid production, they are reliant on sucrose-based feed-stocks [2]. There is an increasing need for a switch to more sustainable fermentation methods that use lower cost substrates and underused resource streams. More efficient strain development procedures are therefore indispensable. Furthermore, *A.* *niger* holds the metabolic potential to convert wide-ranging feedstocks into a plethora of value-added chemicals. Genome sequencing efforts as well as improvements in targeted engineering strategies are making available the tools necessary to develop strains that release this potential.

The different applications of *A.* *niger* in the production of citric acid and enzymes stem from different parent strains; the naturally evolved citric acid producing ATCC1015 strain and the industrial enzyme producing CBS 513.88 strain. These two strains have both been subject to genome studies and their genomes have been sequenced and annotated [3, 4]. This paved the way for genome-scale metabolic modelling of *A. niger*, and to date there are three published genome-scale metabolic models (GSMM) of *A. niger*; iMA871 [5], iHL1210 [6], and more recently iJB1325 [7]. Genome-scale metabolic modelling opens up the possibility to couple genomic and metabolomic information with computational techniques aimed at guiding metabolic engineering strategies. The mathematical method of flux balance analysis (FBA) has been used to make predictions of growth and output of metabolites in response to different conditions, and to probe the effects of gene knock-outs. In previous work, we established a dynamic model of *A. niger* organic acid fermentation, that accurately captures physiological characteristics, providing a more accurate predictive platform to inform engineering strategies [8].

The first GSMMs of *A. niger* were based on the genome annotation of the enzyme producing CBS 513.88 strain. Despite the inclusion of ATCC1015 gene assignments in iMA871, these were based on the CBS 513.88 gene–protein–reaction (GPR) associations. Therefore, the reliability of the ATCC1015 GPR associations in iMA871 is questionable and ATCC1015 genes without a match in the CBS 513.88 strain would not be included. An extensive comparative genomics study highlighted the significant genetic diversity between these strains, revealing around 400 to 500 unique genes in each strain [4]. This genetic diversity may underlie the different roles of the two strains, and prompts the need for an ATCC1015-specific GSMM of *A. niger* relevant to citric acid production. Therefore, in this work we constructed an exhaustive ATCC1015-specific metabolic model of *A. niger*, iDU1756. We employed an annotation process that combined both Blast2GO [9] and KEGG Automatic Annotation Server (KAAS) [10] and used the latest and most complete version of the ATCC1015 genome. This gave an exhaustive list of metabolic reactions, which we used to create more accurate and reliable ATCC1015 GPR associations. From this list, we also searched metabolic reactions not present in previous *A. niger* models for evidence. In doing this work, another *A. niger* GSMM iJB1325 [7] was published, also ATCC1015-specific. Upon comparing iJB1325 with our model, we decided to continue using iDU1756 for further work, primarily because it encompasses the metabolic areas relevant to organic acid production and also due to its greater depth of GPR associations that is important when considering engineering strategies. A detailed comparison of the two models is included in this paper.

The power of FBA models has been underexploited, with most studies focused on simulating gene knock-outs [11–16]. The effects of forcing or constraining flux of metabolic reactions can also be predicted in addition to gene deletion or insertion. This can inform knock-up and knock-down strategies, now that engineering approaches to accomplish these are becoming increasingly available [17, 18]. A metabolic engineering strategy often involves a multitude of targets. Computational approaches can be used to exhaustively evaluate combinations of a small number of targets. On increasing the complexity of combinations however, the resulting combinatorial explosion leads to an enormous search space. Moreover, the incorporation of targets that force or constrain flux to differing degrees significantly expands the search space.

To find optimal solutions in search spaces that are beyond the reach of exhaustive methods requires metaheuristic algorithms. These algorithms are capable of finding optimal solutions without the need for an exhaustive search, making them computationally feasible. One such algorithm is the genetic algorithm (GA) or evolutionary algorithm (EA), that is designed based on natural evolutionary processes, and its applicability for suggesting metabolic engineering strategies has been demonstrated in previous studies focused on the engineering of *Escherichia coli* and *Saccharomyces cerevisiae* for succinate and lactate production [15, 16, 19]. More recently, a framework combining in silico evolution with FBA was applied to capture the long-term evolution of *E. coli* and its adaptive diversification [20]. In this study, we designed a GA for in silico evolution of *A.* *niger* organic acid production, to find solutions that optimise the production of a given acid. Based on our previously developed dynamic model that simulates batch fermentation [8], we derived a fitness function that estimates the rate of production of a target organic acid, using information from parameterised static FBA simulations at chosen time-points of fermentation. The operators of the GA were designed and its parameters tuned to give an optimal evolutionary performance for this application. Once developed, we first employed the algorithm to find predictive solutions that optimise citric acid production. We then demonstrated the full power of the algorithm by successfully evolving production of other organic acids not normally produced, switching acid output from citric to the target acid. The solutions found required complex combinations of mutations, with finely tuned flux bounds, highlighting the importance and value of metaheuristic approaches to solving these problems. The results obtained by use of this algorithm have biotechnological significance, suggesting engineering strategies that could develop *A. niger* strains as production platforms for these various organic acids of commercial value.

## Results

### Assembly of ATCC1015-specific gene–protein–reaction associations

**Fig. 1:**
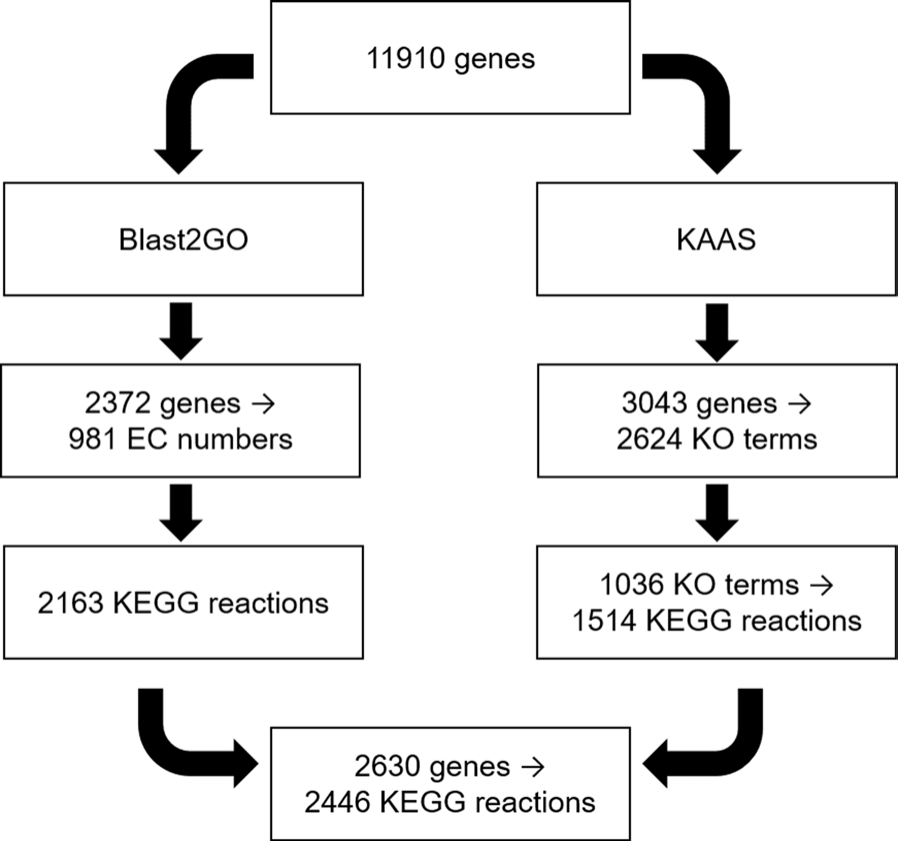
Schematic of annotation process used to map ATCC1015 genes to KEGG reactions

The ATCC1015 strain is the parent citric acid producing strain, and so for a metabolic model of *A. niger* to be relevant to the property of citric acid production it is important that it reflects this strain. The gene assignments in the previous metabolic model of *A. niger*, iHL1210 [6], correspond to the enzyme producing CBS 513.88 strain. The iMA871 model [5] included ATCC1015 gene assignments, however these were secondary and mapped from CBS 513.88 genes using an older version of the ATCC1015 genome annotation. At the time of initiating this work, no reliable ATCC1015-specific model existed, and so to create an ATCC1015-specific model of *A. niger* for this work we employed a reconstruction process (Fig. 1) based on the latest ATCC1015 genome annotation [4]. Available from the Joint Genome Institute, version 4.0 has 11,910 genes compared to 11,197 genes in version 3.0 used in iMA871. The 713 extra genes in the latest annotation explain the missing ATCC1015 gene assignments in iMA871. In the reconstruction process, two annotation tools were used: Blast2GO [9] and KEGG Automatic Annotation Server (KAAS) [10]. Using Blast2GO, 2372 genes were mapped to 981 EC numbers, which were mapped to 2163 KEGG reactions. Using KAAS, 3043 genes were mapped to 2624 KO (KEGG Orthology) terms. 1036 of these KO terms were mapped to 1514 KEGG reactions. From the KAAS output, 1370 genes mapped to 768 EC numbers, and 1340 genes mapped to KEGG reactions. The outputs from Blast2GO and KAAS were compared. 283 of the 1514 KEGG reactions from the KAAS output were not found in the Blast2GO output. 932 of the 2163 KEGG reactions from the Blast2GO output were not found in the KAAS output. The differences are likely due to the Blast2GO output being more exhaustive and the KAAS output being more specific. The two reaction lists were combined into 2446 KEGG reactions from a total of 2630 ATCC1015 genes. 868 of these 2446 KEGG reactions were matched to 994 reactions in the previous *A. niger* model iHL1210. Of the reactions in iHL1210 without a match, 125 were matched to EC numbers and KO terms from the annotation process, bringing the total number of reaction matches to 1119. The corresponding ATCC1015 genes were assigned to these 1119 reactions. BLASTP was used to assign ATCC1015 genes to those reactions in iHL1210 that had no match to the list of KEGG reactions. The gene assignments for 11 of these reactions had no hits above the identity and *e*-value thresholds (90% and 1e−20) (see Additional file 1: Table S1). Two of these had hits within the *e*-value threshold but below the identity threshold, which were included. The other gene assignments were searched against version 3.0 of the ATCC1015 genome annotation, and the gene assignments for two reactions had hits which were included. This resulted in a total of 1729 unique ATCC1015 genes and an intermediate model. This is significantly higher than existing *A. niger* models suggesting more exhaustive GPR associations in this model which act as a valuable resource when considering engineering strategies.

### Evidence-based verification of new metabolic reactions and construction of iDU1756

1578 reactions in the list of KEGG reactions from the annotation process could not be found in previous *A. niger* models and so we searched these in the *A. niger* literature for evidence. Evidence was found for 34 reactions (Table 1). Two additional reactions not present in the KEGG reaction list were added based on literature evidence. One of these (KEGG reaction R01767) completed the amygdalin degradation pathway [21]. The other reaction, the interconversion of galactitol and l-sorbose, filled the missing link in the oxidoreductive galactose catabolic pathway [22, 23]. One reaction (KEGG reaction R02396), the means of mitochondrial acetyl transfer via acetylcarnitine [24, 25], was added to both cytosolic and mitochondrial compartments. A new transport reaction that completes the mitochondrial acetyl transfer pathway via acetylcarnitine was added. These 37 new reactions were added to the model. An additional 27 unique genes corresponding to these new reactions were added, giving a total of 1756 unique ATCC1015 genes and the ATCC1015-specific model iDU1756. An additional ten transport reactions (see Additional file 1: Table S2) and 34 input/output reactions (see Additional file 1: Table S3) were added to iDU1756. The total number of reactions in iDU1756 is 1845. 47 new compounds are included in iDU1756 (see Additional file 1: Table S4), 37 of which are new unique metabolites, bringing the total number of unique metabolites to 939. 1544 reactions in the sequence-based KEGG reaction list were not included in iDU1756 due to the absence of literature evidence. Corresponding to these excluded reactions, there are 1020 unique ATCC1015 genes absent from iDU1756, which have putative metabolic activities. There is therefore the potential for even further expansion of metabolic coverage. We have provided the iDU1756 model in both spreadsheet format (see Additional file 2) and in SBML format (see Additional file 3).

**Table 1:** New reactions in iDU1756 and corresponding literature evidence

KEGG reaction	iDU1756 reaction	Function	Evidence
R06077	CELLUe + H_2_Oe → BDGLCe	Cellulose degradation	[44]
R06101	STACe + H_2_Oe → MNNTe + FRUe	Stachyose degradation	[45, 46]
R06202	GALACTANe + H_2_Oe → GLACe	Galactan degradation	[47]
Not found	GALOL + NAD ⇔ SOR + NADH	Oxidoreductive galactose catabolism	[22, 23]
R00053	D345THBe + H_2_Oe → 345THBe	Digallate degradation	[48–52]
R03372 + R03394	IP6e + 4*H_2_Oe → IP2e + 4*PIe	Phytate degradation	[53, 54]
R02997	CLGe + H_2_Oe → CAFe + QTe	Chlorogenate degradation	[55, 56]
R10040	LNMe + H_2_Oe → ACHe + BDGLCe	Linamarin degradation	[57]
R02156	QCTe + O_2_e → 2PCe + COe	Quercetin degradation	[58]
R02985	AMYDe + H_2_Oe → PRNSe + DGLCe	Amygdalin degradation	[21]
R02558	PRNSe + H_2_Oe → MDNe + DGLCe	Amygdalin degradation	[21]
R01767	MDNe → HCNe + BALe	Amygdalin degradation	[21]
R04103	PCNe + H_2_Oe ⇔ 6APCNe + PHACe	Penicillin G degradation	[59]
R03024	4NPPe + H_2_Oe → 4NPe + PIe	4-nitrophenyl phosphate degradation	[60–64]
R00505	UDPGAL ⇔ UDPGALF	UDP-alpha-d-galactofuranose for galactoglucomannan production	[43]
R01758	LAOL + NAD → LARAB + NADH + H	l-arabitol oxidation	[65]
R09477	XOL + NAD → XYL + NADH + H	Xylitol oxidation	[65]
R02396	ACCOAm + CARm ⇔ COAm + ALCARm	Mitochondrial acetyl transfer	[24, 25]
Transport reaction	CARm + ALCAR ⇔ CAR + ALCARm	Mitochondrial acetyl transfer	[24, 25]
R02396	ACCOA + CAR ⇔ COA + ALCAR	Mitochondrial acetyl transfer	[24, 25]
R00731	TYR + O_2_ → LDOPA + H2O	l-Dopa production	[66]
R00031	O_2_ + 2*TYR → 2*LDOPA	l-Dopa production	[66]
R04300	DPA + H_2_O + O_2_ → DHPHA + NH_3_ + H_2_O_2_	Dopamine metabolism	[67]
R02080	LDOPA → DPA + CO2	l-Dopa metabolism	[66]
R00045	O_2_ + 2*LDOPA → 2*DQ + 2*H_2_O	l-Dopa metabolism	[66]
R01010	T3P2 + H_2_O → GLYN + PI	Glycolytic reaction	[68]
R07253	ACCOA + 3*MALCOA + NADPH → 6MSA + 4*COA + 3*CO_2_ + NADP + H_2_O	6-methylsalicylate production	[69]
R01408	HCN + H_2_O → FMM	Cyanide degradation	[70]
R02943	TRP + DMPP → DMAT + PPI	Tryptophan prenylation	[71]
R01657	DMPP + TRP → PPI + MBT	Tryptophan prenylation	[71]
R05655	PHN + O_2_ + NADH + H → PHNO + H_2_O + NAD	Phenanthrene degradation	[72–75]
R00815	PHL + O_2_ + NADPH + H → CCL + NADP + H_2_O	Phenol degradation	[76]
R01372	PHPYR + O_2_ → 2HPAC + CO_2_	Phenylalanine metabolism	[77]
R01836	TST + NAD → AND + NADH + H	Steroid biotransformation	[78]
R01838	TST + NADP → AND + NADPH + H	Steroid biotransformation	[78]
R01837	DHAND + NAD → AND + NADH + H	Steroid biotransformation	[78]
R07855	PHAN + H_2_O → PHAC + NH_3_	Phenylacetonitrile degradation	[72, 79, 80]

### New substrates in iDU1756

iDU1756 has nine new carbon sources (Table 2), one new nitrogen source (see Additional file 1: Table S7), and two new phosphate sources (see Additional file 1: Table S8). These new substrates were tested by FBA simulations of iDU1756 and confirmed to function as sole substrates. Two of these have empirical evidence; phenol as a carbon source [26] and phytate as a phosphate source [27]; while the rest are hypothetical.

**Table 2:** New carbon sources in iDU1756

iDU1756 compound	KEGG compound	Compound name	Empirical evidence
GALACTANe	C05796	Galactan (extracellular)	Hypothetical
CLGe	C00852	Chlorogenate (extracellular)	Hypothetical
LNMe	C01594	Linamarin (extracellular)	Hypothetical
AMYDe	C08325	Amygdalin (extracellular)	Hypothetical
PRNSe	C00844	Prunasin (extracellular)	Hypothetical
MDNe	C00561	Mandelonitrile (extracellular)	Hypothetical
PCNe	C05551	Penicillin G (extracellular)	Hypothetical
PHLe	C00146	Phenol (extracellular)	[26]
PHANe	C16074	Phenylacetonitrile (extracellular)	Hypothetical

### Comparison of iDU1756 to the latest model iJB1325

We compared the iDU1756 model to the recently published iJB1325 model that is also ATCC1015-specific, to identify any major discrepancies between the two models and to assist future studies aimed at making a consensus and updated model. The metabolites, genes, and reactions of the two models were compared and lists generated of those present in both and those present in only one model or the other (see Additional file 4). Some reactions were comparable but not exactly matched, and in these cases the sources of discrepancy were highlighted. The model comparison is summarised in Table 3. When comparing the two models, we found some concerning discrepancies, such as the lack of fumarate reductase in iJB1325 that we subsequently found to be important to the in silico evolutionary work. We also identified the presence of some reactions in iJB1325 such as cytosolic citrate synthase that have not previously been detected in vivo in *A. niger*. FBA simulations performed with iJB1325 highlighted some additional minor concerns, including phosphate imbalances in some of the reactions that cause conflicting results when simulating phosphate-limited growth important to citric acid production [8], as well as the absence of flux for a key TCA cycle reaction and discrepancies in the reversibility of some reactions. In addition, missing metabolic reactions in iDU1756 captured by iJB1325 were not considered important in our work as we found them to be predominantly inactive (over 95% carrying zero flux) under the conditions imposed in simulations of organic acid fermentation. We conclude that the two models together provide an outstanding *A. niger* resource and that the construction of iDU1756 was a necessary step for performing large-scale in silico evolutionary modelling.

**Table 3:** Comparison of iDU1756 with iJB1325 (see Additional file 4)

	In both iDU1756 and iJB1325	In iDU1756 and not in iJB1325	In iJB1325 and not in iDU1756
Total metabolites	1147	174	671
Unique metabolites	851	159	549
Total metabolites not present in other compartments in other model	N/A	109	486
Unique metabolites not present in other compartments in other model	N/A	95	367
Genes	1022	734	303

	Exact match in other model	Discrepant match in other model	No match in other model
Reactions in iDU1756	764	750	348
Reactions in iJB1325	748	800	772

### Application and parameterisation of iDU1756 for organic acid fermentation based on dynamic modelling

We used our new model iDU1756 in FBA simulations of citric acid production, using parameters from our previously described dynamic model of *A. niger* citric acid fermentation [8]. The biomass composition used is shown in Additional file 1: Table S9, which is largely the same as other models with some modifications based on previous fitting to empirical data [8]. The non-growth-associated maintenance (NGAM) was set to 1.9 mmol gDW^−1^ h^−1^, in accordance with iMA871, as this parameter was applied in our previous fitting of the dynamic model [8]. Acid dissociation reactions were included in iDU1756, for modelling of organic acid production driven by proton production [28]. The input/output fluxes corresponding to the two growth phases, phosphate storage and proton production, are shown in Table 4. These give a more accurate prediction of citric acid output with relevance to a batch fermentation setting compared to previous models which were based on citric acid being produced as a by-product during growth or forcing citric output at a fixed value [5, 6]. In this case, citric acid is produced for the objective of proton production during phosphate-limited growth. We applied this parameterised iDU1756 model for the in silico evolution of organic acid production, using a fitness function that encompasses findings from dynamic modelling and estimates the rate of organic acid production in a computationally efficient manner without the need for extensive dFBA.

**Table 4:** Input/output fluxes in iDU1756

Input/output reaction	Flux during phosphate storage (mmol h^−1^ gDW^−1^)	Flux during proton production (mmol h^−1^ gDW^−1^)
Glucose (DGLCe ⇔)	− 0.445	− 0.317
External phosphate (PIe ⇔)	− 0.0144	0.0
Internal phosphate (PI ⇔)	0.0124	− 7.43E−4
Oxygen (O_2_e ⇔)	− 0.974	− 0.552
Protons (Hpe ⇔)	0.0	0.0062
Citric acid (CIT-e ⇔)	0.0	0.162
Biomass	0.0435 (gDW h^−1^)	0.0159 (gDW h^−1^)

### In silico evolution of citric acid production

**Fig. 2:**
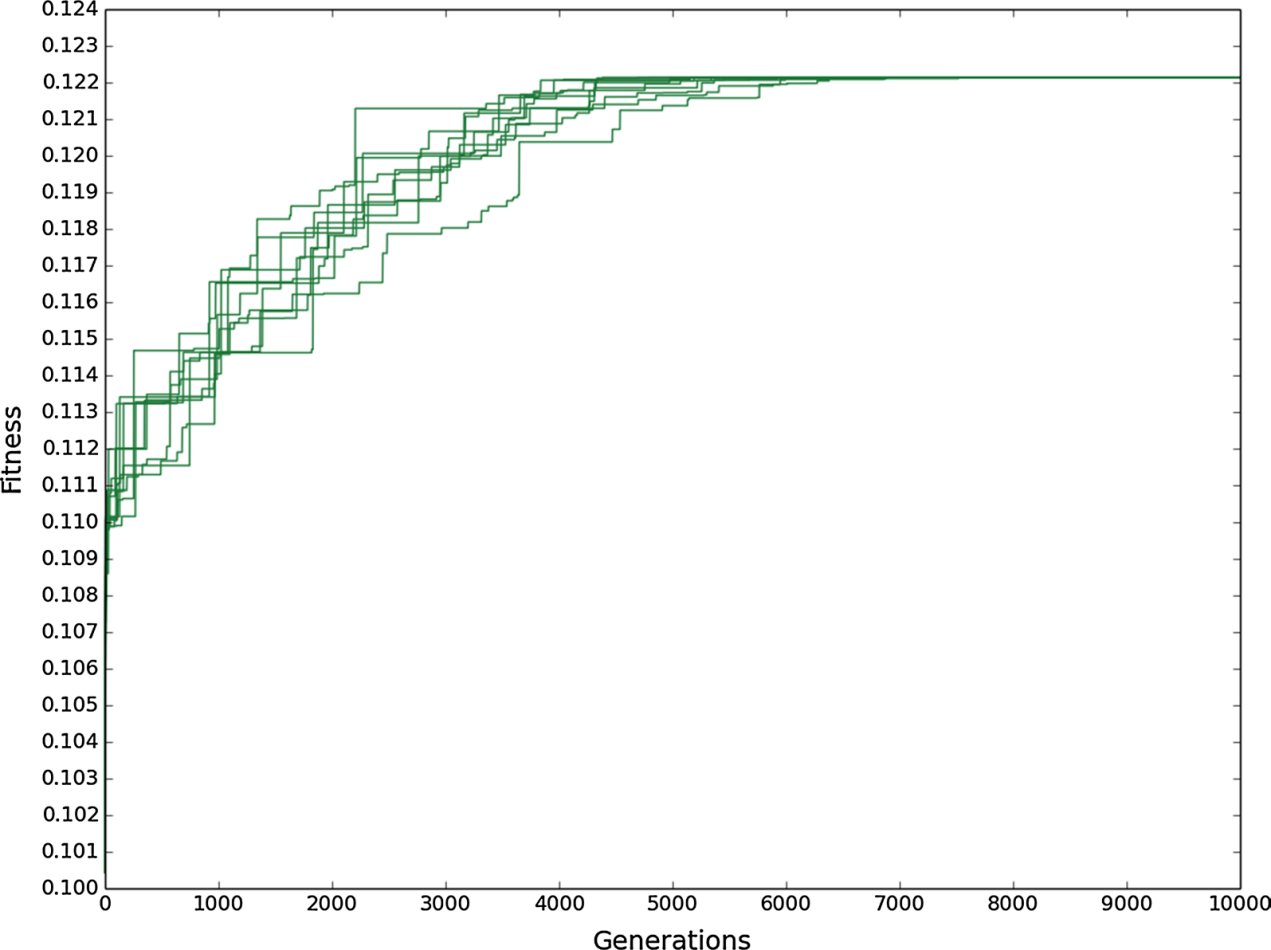
Increase in highest population fitness over generations with evolutionary pressure towards citric acid production. Each line corresponds to the evolutionary course of one replicate run

To perform in silico evolution of organic acid production, we designed a genetic algorithm (see “Methods”) and first applied this to predict changes that optimise citric acid production with glucose as substrate. 10 replicate runs were performed. The initial fitness (wild-type) was 0.1 and increased by only 20% to around 0.12 over the course of 5000 generations (Fig. 2). Evolution was continued up to 10,000 generations, however, no further increases in fitness occurred. The variation in evolutionary speed between replicate runs was very low, with all runs achieving the same maximum fitness in an average of 4092 generations and standard deviation of 556 generations.

**Fig. 3:**
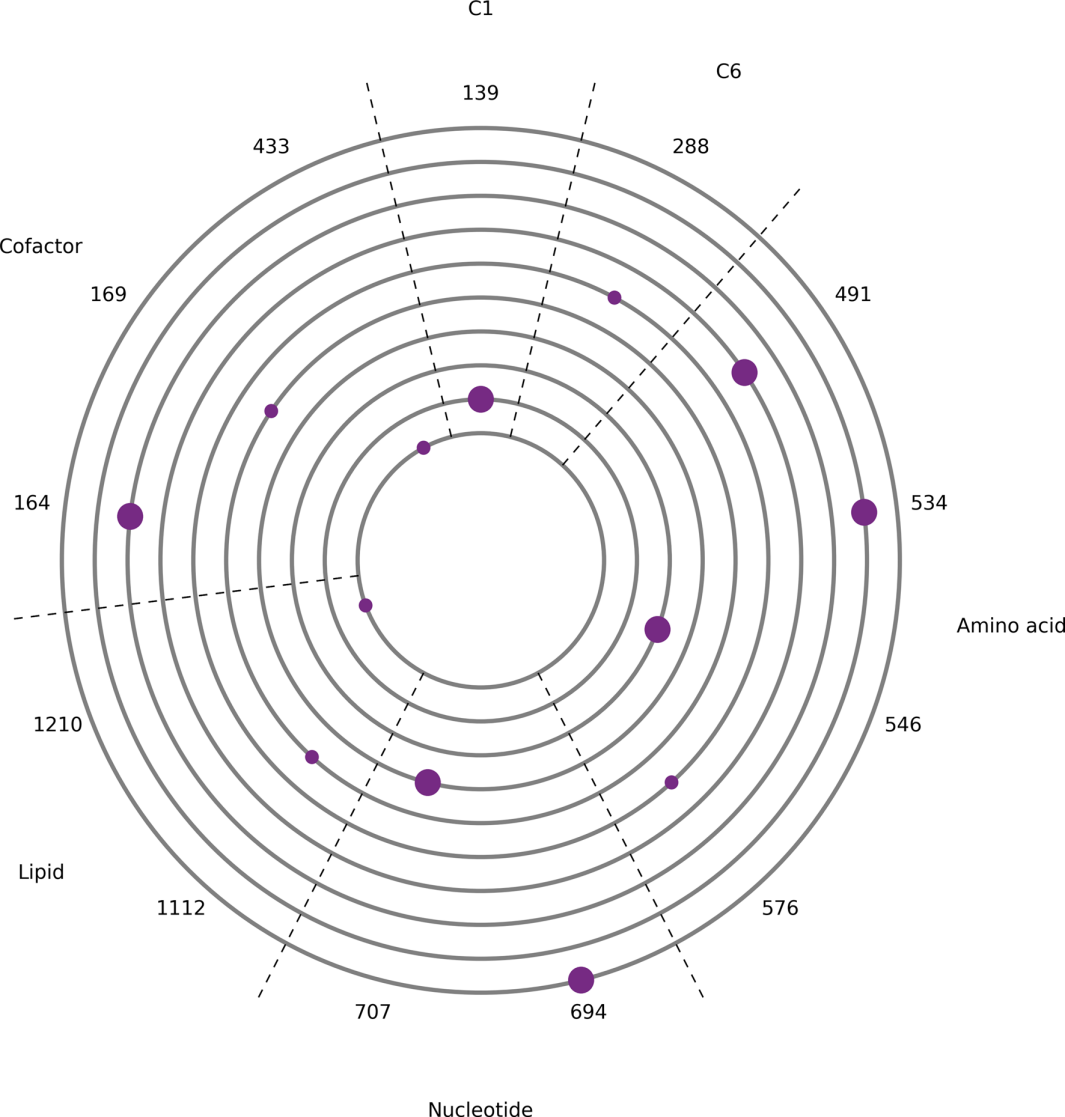
Evolution plot showing the site and frequency of mutations from 10 independent runs with evolutionary pressure towards citric acid production. Each of the ten grey circles corresponds to the results of one replicate run. The numbers on the outside are indices and refer to reactions where mutations occurred. The corresponding reactions are given in Additional file 5: Table S15. Dots on the grey circles align with these indices and indicate where mutations occurred. The diameter of each dot is proportional to the frequency of the corresponding mutation across solutions from the run. The sectors indicate areas of metabolism that the mutations targeted

The evolution output was analysed and the site and frequency of mutations are indicated in Fig. 3. Each run only gave one or two solutions, each having a single mutation that targeted growth. The mutations across different solutions were found to be undirected and scattered across different areas of metabolism, constraining production of different biomass components. No same mutation occurred in more than one run, suggesting a large number of possible solutions resulting from multiple growth targets that are independent from the metabolic reactions required in citric acid production.

One solution was chosen (see Additional file 1: Table S10), and applied in dynamic modelling of organic acid fermentation with comparison to the wild-type (see Additional file 6: Figure S2). Growth clearly became further constrained in the mutant upon inducing mutations at the point of phosphate depletion, with an 18% drop in total biomass produced and a 40% increase in the time taken for growth to finish. Between days 4 and 5.5, the rate of citric acid production was 62.5% higher, and the total produced was 118% of the wild-type.

### In silico evolution of succinic acid production

**Fig. 4:**
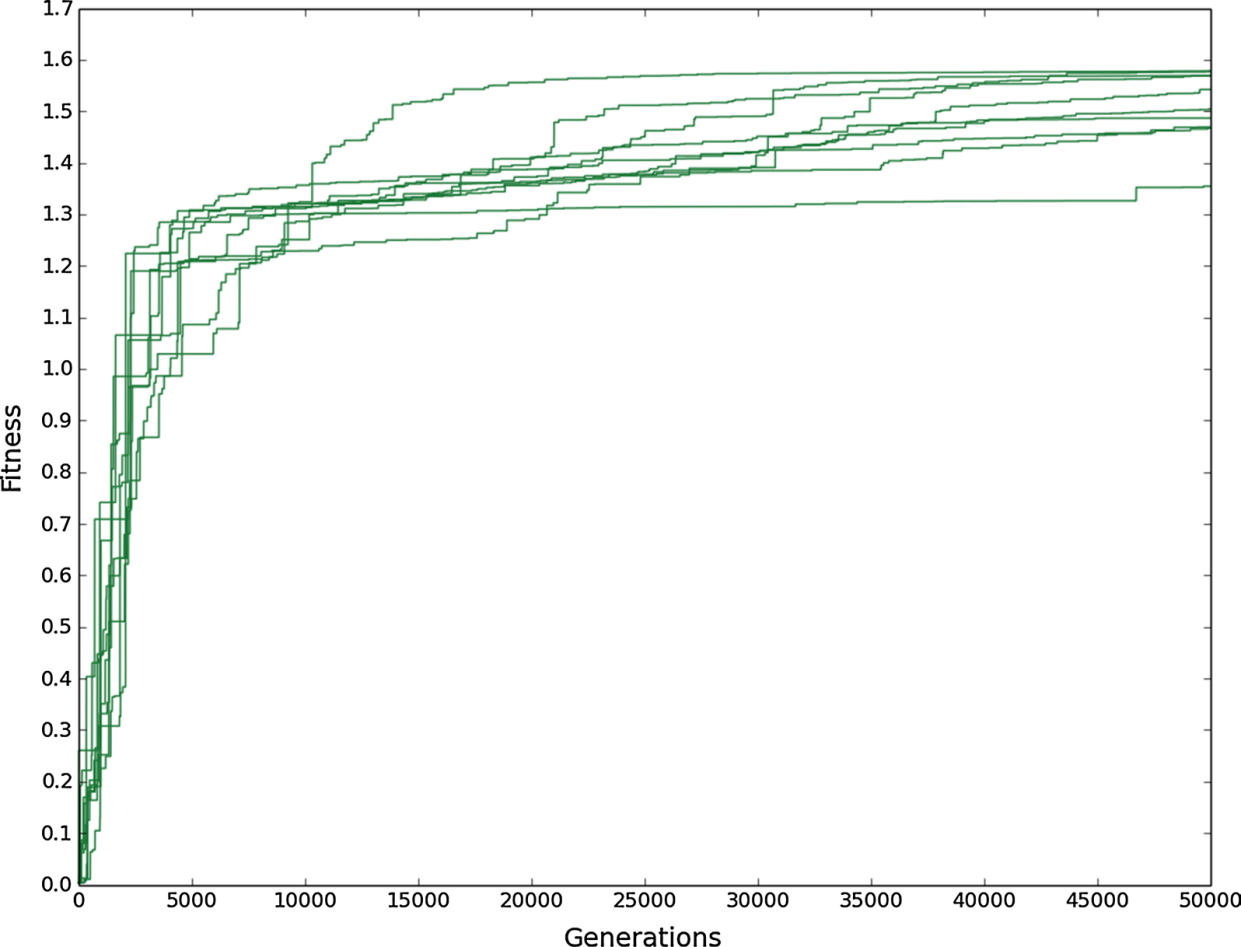
Increase in highest population fitness over generations with evolutionary pressure towards succinic acid production. Each line corresponds to the evolutionary course of one replicate run

We applied the genetic algorithm to predict changes that switch acid output from citric to succinic and optimise succinic acid production with glucose as substrate. 10 replicate runs were performed for a duration of 50,000 generations each. Succinic acid production successfully evolved, with a rapid increase in fitness in the first 10,000 generations followed by a more gradual increase up to 40,000 generations, after which it appeared to plateau (Fig. 4). To be more confident that the maximum fitness was reached by 50,000 generations, one run was performed up to 100,000 generations (see Additional file 6: Figure S3). The fitness was constant after 40,000 generations suggesting no further increases were possible. The maximum fitness reached varied between replicate runs by up to 14% of the highest maximum fitness, suggesting a slower evolutionary speed in some cases or trapping at less optimal solutions. 40% of the runs achieved the same maximum fitness and were constant after 40,000 generations, suggesting these reached the global optimum.

**Fig. 5:**
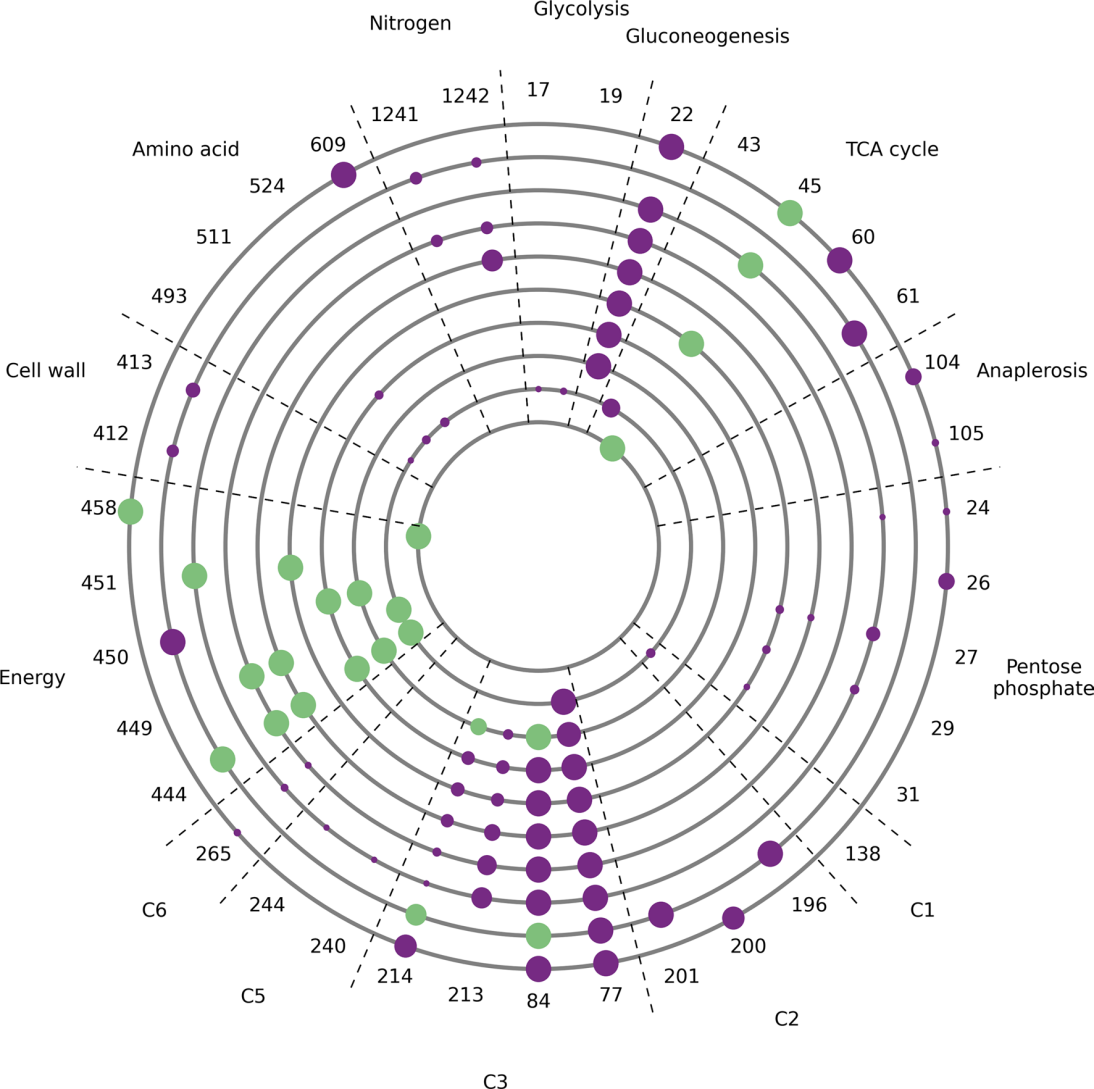
Evolution plot showing the site and frequency of mutations from 10 independent runs with evolutionary pressure towards succinic acid production. Each of the ten grey circles corresponds to the results of one replicate run. The numbers on the outside are indices and refer to reactions where mutations occurred. The corresponding reactions are given in Additional file 5: Table S15. Dots on the grey circles align with these indices and indicate where mutations occurred. The diameter of each dot is proportional to the frequency of the corresponding mutation across solutions from the run. A frequency cut-off of 0.2 was applied. Mutations with a frequency lower than the cut-off are not represented. Green dots indicate mutations that when complemented decrease target acid flux by > 95%. Purple dots indicate mutations that when complemented decrease target acid flux by < 95%. The sectors indicate areas of metabolism that the mutations targeted

The evolution output was analysed and the site and frequency of mutations are indicated in Fig. 5. Nine mutations had frequency over 70% and were repeated across replicate runs, and targeted four distinct areas of metabolism (energy, C3, TCA cycle, and gluconeogenesis). In particular, the activities of ubiquinol oxidase, succinate dehydrogenase (ubiquinone), pyruvate decarboxylase, d-lactate dehydrogenase, and pyruvate carboxylase were constrained.

**Fig. 6:**
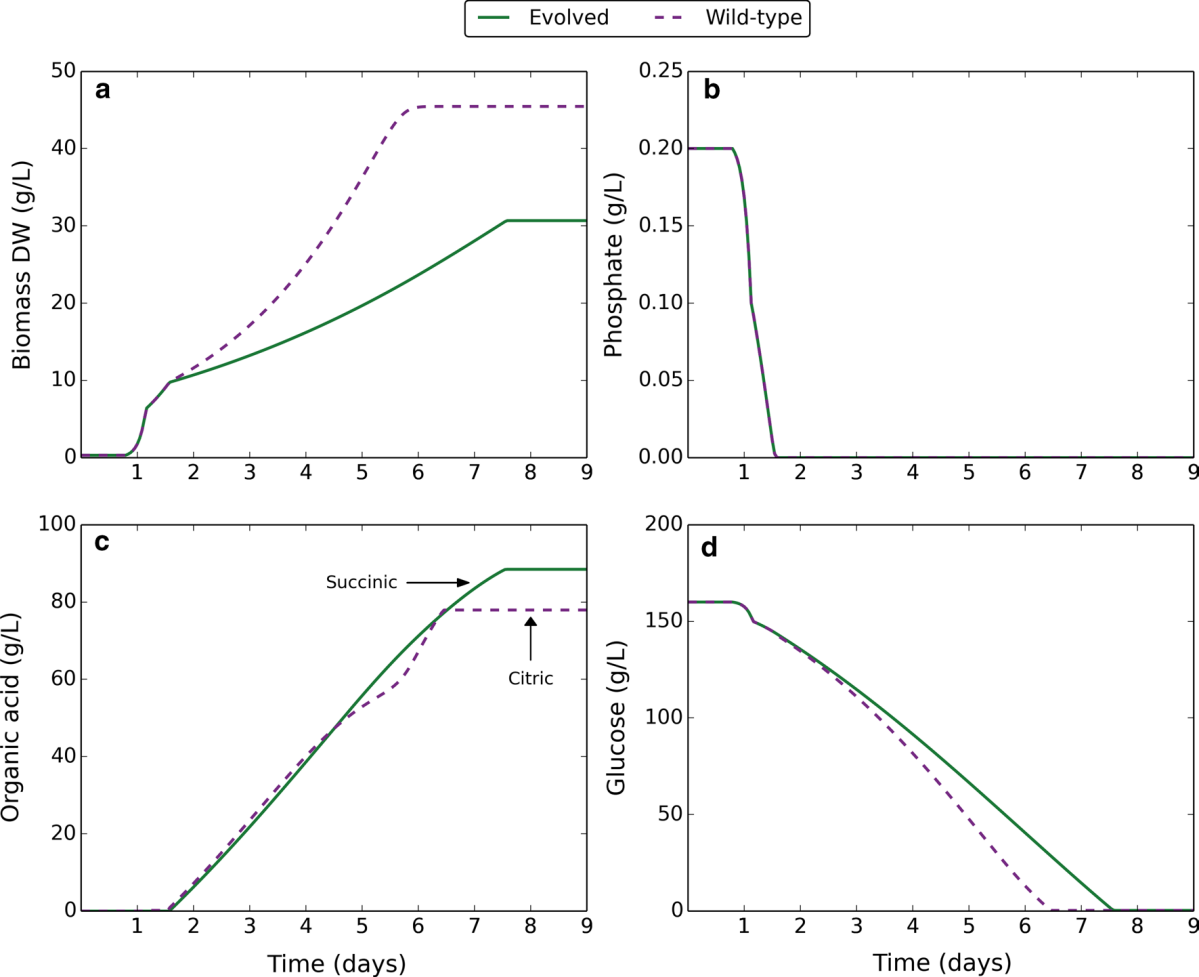
Dynamic modelling of organic acid fermentation comparing the wild-type with a solution from in silico evolution towards succinic acid production. Green solid lines correspond to an evolved succinic acid producer, using a solution that best represents the average and based on fitness (Table 6). Purple dashed lines correspond to the wild-type. Mutations were induced at the point of external phosphate depletion. **a** Change in biomass dry weight (g/L) over time. **b** Change in external phosphate concentration (g/L) over time. **c** Change in external organic acid concentration (g/L) over time. Lines are annotated to indicate the organic acid produced. **d** Change in external glucose concentration (g/L) over time

The solution that best represented the average and with fitness near the maximum was chosen (Table 5), and applied in dynamic modelling of organic acid fermentation (Fig. 6) with mutations active beyond the point of phosphate depletion. Acid output was completely switched from citric to succinic in the evolved succinic producer, with a predicted yield of 90 g/L succinic acid. Growth was also more constrained than in the wild-type, resulting in a higher rate of acid production. It was also observed that succinic acid was produced as a by-product during growth optimisation, and was not dependent on the proton production objective function. The mutations therefore placed flux constraints that forced production of succinic acid to achieve optimal growth.

**Table 5:** Example solution from evolution of succinic acid production

Index	Reaction	Mutation effect	Complementation results		
			% Fitness decrease	% Acid flux decrease	% Growth increase
444	QH_2_m + 0.5*O_2_m → Qm + H_2_Om	UC	99.8	100	98.2
449	QH_2_m + 2*FERIm + 2*Hm → Qm + 2*FEROm + 4*Ho	UC	99.8	100	98.2
213	PYR + NADH + H → LLAC + NAD	UC	85.3	86.2	19.3
84	LAC + NAD ⇔ PYR + NADH + H	LC	85.2	86.2	19.3
77	H + PYR → ACAL + CO_2_	UC	77.1	77.8	9.6
265	GLCNT + ATP → D6PGC + ADP + H	UC	52.4	54.4	12.5
24	G6P + NADP → D6PGL + NADPH + H	UC	49.7	51.8	12.3
22	ATPm + PYRm + H_2_Om + CO_2_m → ADPm + PIm + OAm + 2*Hm	UC	18.9	22.5	14
1242	NH_4_OH ⇔ NH_3_ + H_2_O	UC	12.8	13.8	3.5

The example solution is chosen as the best representative of the average solution and based on fitness. The mutation effect is given as UC or LC. UC corresponds to a mutation that imposes a flux constraint on the upper bound. LC corresponds to a mutation that imposes a flux constraint on the lower bound. Complementation results are given for each mutation, showing the effect on fitness, target acid flux, and growth when the mutation is complemented with the wild-type while retaining the other mutations

### In silico evolution of lactic, malic, acetic, and gluconic acid production

In addition to succinic acid, we also applied the genetic algorithm to predict changes that switch acid output from citric to lactic, malic, acetic or gluconic acid and optimise production of the target acid with glucose as substrate. These were done similarly to in silico evolution of citric and succinic acid production, with 10 replicate runs performed for each. 30,000 generations were needed to evolve production of the acids lactic, acetic and gluconic, while 50,000 generations were needed for malic acid. The fitness versus generation plots for the in silico evolution of these acids are shown in Additional file 6: Figures S4, S7, S10, and S13, respectively.

The evolution outputs corresponding to these acids were analysed and the site and frequency of mutations are indicated in the evolution plots provided in Additional file 6: Figures S5, S8, S11, and S14.

Solutions from in silico evolution of the production of these acids that best represented the average and with fitness near the maximum are given in Additional file 1: Tables S11–S14. These were applied in dynamic modelling of organic acid fermentation similarly to citric and succinic, and the results can be seen with comparison to the wild-type in Additional file 6: Figures S6, S9, S12, and S15. In each case, acid output was completely switched from citric to the target acid in the evolved producers.

## Discussion

In this study, we employed our previously developed dynamic modelling framework [8] coupled with a genetic algorithm to evolve the production of various organic acids in silico. We used a fitness function that encapsulates the dynamic modelling in a computationally efficient manner, to estimate the rate of production of a given organic acid in a setting relevant to batch fermentation. As the output of this work is target suggestions for strain engineering, we paid particular attention to the strain-specificity of the underlying metabolic model and its GPR associations, as these are an essential resource when considering genetic engineering strategies to target particular metabolic reactions. This highlights the value of our exhaustive ATCC1015-specific GPR in iDU1756 which is complemented by the iJB1325 model also based on the ATCC1015 strain. The two models provide an in-depth resource for identification of genetic information and give targets for future studies to further elucidate the metabolic coverage provided by *A. niger*.

By incorporating findings from dynamic modelling of *A. niger* organic acid fermentation [8], we developed iDU1756 into a purpose-built model of organic acid production. We then designed and optimised a genetic algorithm for in silico evolution of *A. niger* organic acid production using a fitness function that estimates the rate of production of the target organic acid, informed by parameterised static FBA simulations of chosen fermentation time-points. The dynamic modelling framework enabled the targeting of particular phases of growth, notably before the onset of acid production when phosphate is in excess and during acid production when phosphate is limiting. This gave rise to a non-trivial fitness function that represents a compromise between the simplicity and speed of a static FBA simulation and the complexity of a full dynamic simulation at each fitness evaluation.

To establish a picture of the underlying flux changes in solutions from in silico evolution, the flux patterns were examined for each of the evolved target acids. In the case of evolution of citric acid production, the solutions were simply single growth targets with flux constraints that result in a growth rate that gives optimum citric acid productivity. Switching acid output required more complex solutions. As well as inducing a re-distribution of flux that brings about a complete switch of acid output to the target acid, these solutions also constrained growth to give optimum productivity. Our predictions reveal the balance between targeted acid output and biomass production that is only possible to predict by the use of our informed dynamic modelling approach. The importance of this was highlighted by a previous study that investigated the effect of varied phosphate concentration on biomass and citric acid production and the resulting productivity [29]. The authors of this study concluded that optimum productivity requires a balance between growth and product formation that can be achieved by adjustment of the phosphate concentration. In our previous work [8], we showed that the growth rate during the citric acid producing phase is phosphate-limited and determined by the rate of release of stored phosphate. Elucidation of the mechanisms involved would provide additional engineering targets for fine-tuning the rate of biomass production during this phase to suit optimal productivity. The growth targets suggested by our in silico methods in this work would need careful examination prior to in vivo engineering as these could have unpredictable effects causing altered biomass composition rather than constrained growth due to a limitation of metabolic modelling in accurately reflecting the dynamics of biomass production.

When investigating the flux patterns of evolved lactic, acetic, succinic, malic and gluconic acid producers, a relationship was observed for the first three acids with the common feature of disrupted NADH recycling by aerobic respiration, forcing an alternative means of NADH recycling to maintain a high glycolytic flux. This was found to be essential to production of these acids, and was usually caused by mutations targeting both ubiquinol-cytochrome *c* reductase and alternative oxidase. Other mutations frequently occurred that caused a decreased flux through the electron transport chain, targeting core reactions such as ATP synthase and ADP/ATP translocase, and all solutions for these three acids shared the need to block NADH recycling via production of the alternate acids to achieve optimal productivity of the target acid. Recently, a transcriptomics study was performed for the optimised citric acid-producing industrial strain YX-1217 with comparison to the wild-type strain ATCC1015 [30]. This study showed that optimal citric production is associated with heightened activity of the electron transport chain and up-regulation of NADH oxidase-related genes which serve to recycle NADH by aerobic means. Our in silico findings indicate these as key targets for switching acid output from citric to other acids that are products of alternative NADH recycling pathways. It is clear from our work that organic acids production is strongly coupled to the recycling of NADH via various pathways and a good understanding of NADH/NAD^+^ redox dynamics is valuable. A relevant study in this field recently reported the establishment of a system for in vivo monitoring of the cytosolic NADH/NAD^+^ ratio in *Ustilago maydis* under conditions relevant to biotechnology [31]. This allowed the effects of various perturbations on the redox state to be closely monitored.

The subsequent changes in flux patterns of the evolved producers of the acids lactic, acetic, and succinic differed in complexity, with lactic being the simplest case where lactate dehydrogenase provided the sole source of lactic output with concomitant NADH recycling. Lactic acid production in *A. niger* has been previously targeted in two studies by overexpression of lactate dehydrogenase [32, 33] which in one case resulted in lactic acid production up to 7.7 g/L with 13% conversion from glucose, however, no lactic acid was produced when the medium was changed to one typically used for citric acid production [32]. In the two studies citric acid production was either unchanged [32] or even increased [33] upon overexpression of lactate dehydrogenase. These findings are in line with our own that show the necessity of targeting multiple steps to bring about a switch in acid output and the need to block competing NADH recycling pathways. A further study targeted *Aspergillus brasiliensis*, a close relative of *A. niger*, for lactic production via overexpression of lactate dehydrogenase and achieved up to 32.2 g/L lactic acid with 44% conversion from glucose [34], however, this yield of acid is still relatively low compared to that typically achieved for citric acid production. The outcomes of these simplistic engineering approaches and the complexity of our in silico work highlight the value of in silico guided engineering for achieving optimal production of the target acid, and this is further demonstrated by the more complex changes in flux patterns observed for evolved acetic producers, and yet more complex for succinic. For acetic the breakdown of TCA cycle intermediates including citrate and oxaloacetate was the primary source, and this involved the recycling of the breakdown product oxalate into formate and then formaldehyde, which was fed back into glycolysis requiring the functioning of the pentose phosphate pathway. The action of formaldehyde dehydrogenase enabled the re-supply of NAD. The competing patterns of different solutions to acetic production employing alternative pathways with varying carbon usage and alternate means of NADH recycling led to the emergence of differing local maxima in the evolutionary optimisation for this acid. Microbial production of this acid is typically performed by acetic acid bacteria via oxidation of ethanol [35], and to our knowledge no attempts have been made to engineer *A. niger* for acetic acid production. The flux patterns of acetic acid producers evolved in silico give a picture as to how the metabolism would operate for *A. niger* if it were to be developed for optimal acetic production.

**Fig. 7:**
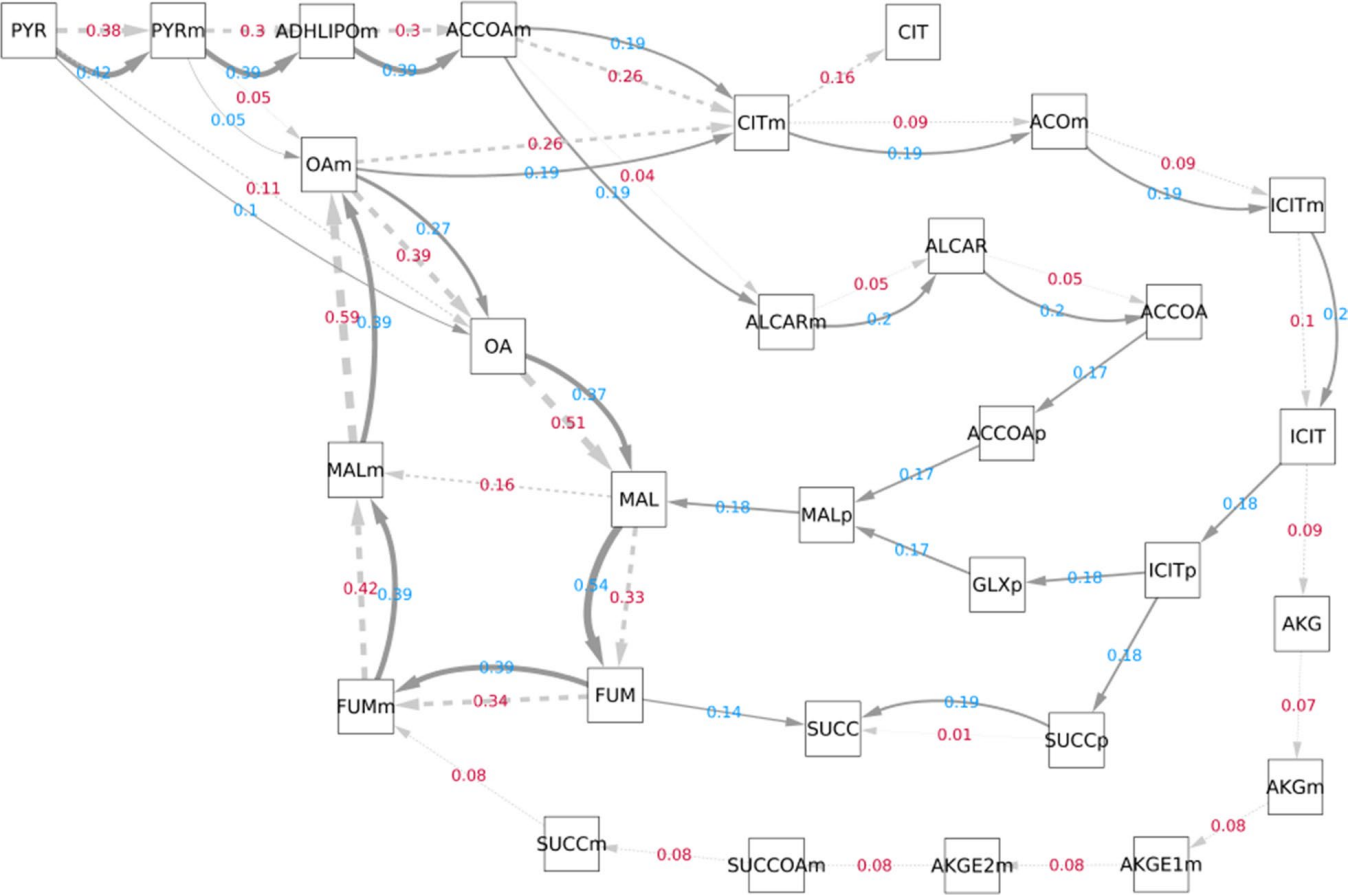
Flux diagram illustrating re-distribution of flux in evolved succinic acid producer compared to wild-type. The chosen solution from evolution of succinic acid production was applied in FBA simulations to determine the re-distribution of flux relative to the wild-type. Flux values are given to two decimal places. Dark grey and solid arrows with light blue flux labels correspond to the evolved succinic acid producer. Light grey and dashed arrows with red flux labels correspond to the wild-type. The thickness of arrows is proportional to the corresponding flux

The solutions that optimise succinic acid productivity appeared to be more complex than those for the other acids, and this is resembled by the more elaborate re-distribution of flux observed in evolved succinic acid producers (Fig. 7). Examination of the flux patterns revealed succinic output to be sourced from two reactions; 56% from isocitrate lyase and 44% from fumarate reductase. Succinic acid production by *A. niger* has been targeted in a previous study [36] where the overexpression of isocitrate lyase and inhibition of succinate dehydrogenase were investigated—our findings illustrate that these are potentially necessary but insufficient to induce succinic production. It was also proposed in this study that fumarate reductase has low activity; we found heightened fumarate reductase activity to be essential for optimal succinic production. Operation of the glyoxylate shunt was responsible for directing flux towards succinic production, via the actions of isocitrate lyase and malate synthase. The conversion of malate to fumarate provided the substrate for fumarate reductase to produce the remaining succinic. The optimal solution required a balance between the two succinic-producing reactions; isocitrate lyase and fumarate reductase. The activity of fumarate reductase provided the alternative means of NADH recycling (via the recycling of FADH_2_ to FAD) upon constraint of the electron transport chain. Fumarate reductase has been targeted previously to enhance succinic acid production in *Aspergillus saccharolyticus*, a species which naturally secretes small quantities of this acid [37]. Expression of NADH-dependent fumarate reductase from *Trypanosoma brucei* increased succinic production from 3.8 to 16.2 g/L while negatively affecting malic and citric acid production [37]. When the same fumarate reductase gene was expressed in an engineered strain of *Aspergillus carbonarius* lacking the glucose oxidase gene the acid production profile was unchanged, yet increased succinic production up to 16 g/L when a C4-dicarboxylate transporter was overexpressed [38]. The studies highlight the need to consider organic acid transport in organisms that do not naturally secrete the target acid. Although successful, these studies only made an initial step to achieving optimal succinic acid production in *Aspergillus*, and the targeting of many other steps as shown by our in silico evolved producers would be necessary to fully realise this goal.

Malic and gluconic acid production evolved differently to lactic, acetic and succinic as there was no need for disrupted NADH recycling by aerobic respiration. Malic acid production evolved primarily through a partial disruption of citrate synthase, which all solutions shared in common, and could be achieved via a specific constraint on citric production as malic production is the next most efficient means of acidification in its absence. A notable study reported recently successfully engineered *A. niger* ATCC1015 to produce significant quantities of malic acid via the targeting of four steps using a Cre-*lox*P based gene editing system [39]. The knockout of oxaloacetate hydrolase together with the overexpression of pyruvate carboxylase, malate dehydrogenase and a C4-dicarboxylate transporter achieved 120 g/L malic acid in shake flask culture and 200 g/L malic acid in fed-batch fermentation [39]. Although the yield of the target product was very high in the engineered strain, citric acid was still produced at 28 g/L showing that a complete switch of acid output had not occurred. Furthermore, the results were obtained under conditions of pH control by addition of CaCO_3_ and may not be reproduced if pH were allowed to drop. This study demonstrated that the engineering of wild-type *A. niger* at multiple steps is entirely feasible, and under the guidance of the in silico findings in this work could potentially achieve optimum productivity of the target product and a complete switch in acid output. In the case of gluconic acid, commercial production with *A. niger* is already established but relies on the action of extracellular glucose oxidase therefore is dependent on the maintenance of a suitable pH [40]. To achieve intracellular production that can operate at low pH our evolutionary simulations predicted the disruption of glycolysis was necessary, causing a by-passing re-distribution of flux via gluconate and forced gluconate accumulation upon constraint of its catabolic pathways. We are not aware of any engineering attempts to re-wire the metabolism of *A. niger* for optimum gluconic acid production via the intracellular route, but our in silico findings suggest that it could be achieved demonstrating the metabolic flexibility and potential of this organism.

Although our work is based on a dynamic model of metabolism we have chosen not to include explicit transport mechanisms, including glucose and phosphate import and product and by-product export, as possible mutable targets. This is a crucial area for future research: the need to up-regulate organic acid export has been highlighted by numerous studies. For example, succinic acid export has been identified as a limiting factor [41] for succinic acid production and overexpression of citrate export in *A. niger* ATCC1015 increased citric production as much as fivefold [42]. These are important considerations when designing a strategy to successfully engineer *A. niger* to produce organic acids, but their incorporation into an in silico evolutionary scheme presents considerable computational difficulties. Further work understanding transport mechanisms in *A. niger* is essential to gain further insight to facilitate the feasibility of computational approaches in the future.

## Conclusions

This work demonstrates how a detailed genome-scale metabolic model (GSMM) can be combined with state-of-the-art dynamic modelling and metaheuristic evolutionary algorithms to provide detailed target suggestions for metabolic engineering in an industrially important organism. We base our work on an extensive GSMM that complements the genomic coverage of previous models and enables a full exploration of this important industrial organism. To devise an appropriate fitness target for this organism growing in batch culture, we constructed a fitness function based on the mathematical analysis of a previously introduced dynamic modelling framework—evaluation of such a dynamic model at each evolutionary time-step would not be computationally feasible, by several orders of magnitude, compared to static FBA evaluations. Our evolutionary framework enabled continuous adjustment of flux bounds suggesting strategies for more complex synthetic interventions beyond simple Boolean gene deletions. The validation of these in vivo would be an important step forward demonstrating the power of in silico guided approaches. Initial experiments should focus on transport of the target acids as previous research shows this to be of primary importance. With appropriate organic acid export functionality in place, steps should be taken to re-wire the metabolism to match that of the evolved in silico producers reported in this work. Flux patterns should be evaluated in silico to identify key steps where high flux is required for optimum productivity, and these should be checked in vivo for potential bottlenecks that could be resolved by targeted overexpression. Many of our in silico target suggestions involve multiple finely tuned constraints to induce the necessary re-distribution of flux, and to achieve these in vivo would require targeted knock-down, promoter engineering, or replacement of native target genes with ones controlled by inducible expression systems. Further efforts are needed to enhance the engineering tool-kit to facilitate such endeavours. This framework for guiding rational engineering, based on the dynamic batch growth found more commonly in industrial processes, offers considerable potential for future investigations focused on the engineering of other organisms to produce desired products.

## Methods

### Assembly of ATCC1015-specific gene–protein–reaction (GPR) associations

To assemble comprehensive and reliable GPR associations specific to ATCC1015, the 11910 ATCC1015 genes of the latest ATCC1015 genome annotation from the Joint Genome Institute (v 4.0) [4] were mapped to KEGG reactions. This was achieved by the use of two annotation tools; Blast2GO [9], and KEGG Automatic Annotation Server (KAAS) [10]. Blast2GO version 2.7.2 was used, and BLASTP was performed using the non-redundant (nr) protein database from the NCBI. EC numbers were assigned to ATCC1015 genes, and these were mapped to KEGG reactions. The ATCC1015 genes were also assigned KO terms by KAAS, and these were mapped to additional KEGG reactions. The two lists of KEGG reactions were combined. The resulting reaction list was searched against reactions in the previous model iHL1210 to identify existing reactions and new reactions based on sequence information. Reaction matches were used to assign ATCC1015 genes. iHL1210 reactions without a match to the list of KEGG reactions were assigned ATCC1015 genes either by matching EC numbers and KO terms or by BLASTP of the CBS 513.88 genes in iHL1210. Thresholds of 90% identity and e-value 1e-20 were used for the comparison.

### Evidence-based verification of new metabolic reactions

Reactions from the list of KEGG reactions that were not found in the previous model were searched for evidence in an exhaustive body of literature using a custom-built automated text search approach in Python. The body of literature contained all records with *Aspergillus niger* or *A. niger* in the title, obtained from Web of Science. The full text was searched if available through the University of York Library or open access. The abstract was searched if the full text could not be accessed, and the title was searched if the abstract was unavailable. Reactions were searched for evidence in each record by using an exhaustive list of search terms for each compound. To increase specificity, common compounds were not searched (KEGG compounds: C00001, C00002, C00003, C00004, C00005, C00006, C00007, C00008, C00009, C00010, C00011, C00013, C00014, C00016, C00027, C00080). Compound search terms were compiled from the following databases: KEGG, MMCD, HMD, PubChem, ChEBI, PDB-CCD, 3DMET, NIKKAJI, KNApSAcK, LIPIDMAPS, and LipidBank. To increase specificity, search terms less than three characters were not included. The percentage of reaction compounds found of those searched and number of search hits were recorded and used to rank the results. Reactions with 60% or more compounds found were manually checked for evidence in records with search hits. Reactions with evidence were added to the model.

### Changes in iDU1756 to reactions from iHL1210

In constructing the iDU1756 model, some changes were made to reactions from iHL1210. The reaction that produces galactoglucomannan was altered to require UDP-alpha-d-galactofuranose based on literature evidence [43]. The coefficients of UDP-galactose and UDP-glucose were changed from 0.435 to 0.332 and from 0.13 to 0.1, respectively, and the coefficient of UDP-alpha-d-galactofuranose was set to 0.133. UDP-alpha-d-galactofuranose is a new compound in iDU1756 and produced by isomerisation of UDP-galactose, a new reaction in iDU1756. This new reaction is essential to biomass production, to produce the galactoglucomannan component of the cell wall, and therefore is a new growth target in iDU1756. Some errors were found in reactions from iHL1210, and corrections were made either to reaction species (see Additional file 1: Table S5) or compartmentalisation (see Additional file 1: Table S6).

### Comparison of iDU1756 to the latest model iJB1325

To perform a detailed comparison and identify discrepancies between iDU1756 and the recently published iJB1325 model, we compared the metabolites, genes, and reactions of the two models and generated lists of those present in both and those present in only one model or the other (see Additional file 4). The sources of discrepancy were highlighted for those reactions comparable but not exactly matched, and Table 3 summarises the comparison. To be able to implement the iJB1325 model for comparative purposes, we had to resolve some minor errors including phosphate imbalances in the reactions r1266 and RNApolym and a compartmentalisation error in the TCA cycle reaction r36c that caused it to carry zero flux, as well as correcting some reactions to be irreversible such as r35 and removing cytosolic citrate synthase (r31) that does not exist in vivo in *A. niger*.

### Designing of genetic algorithm for in silico evolution

**Fig. 8:**
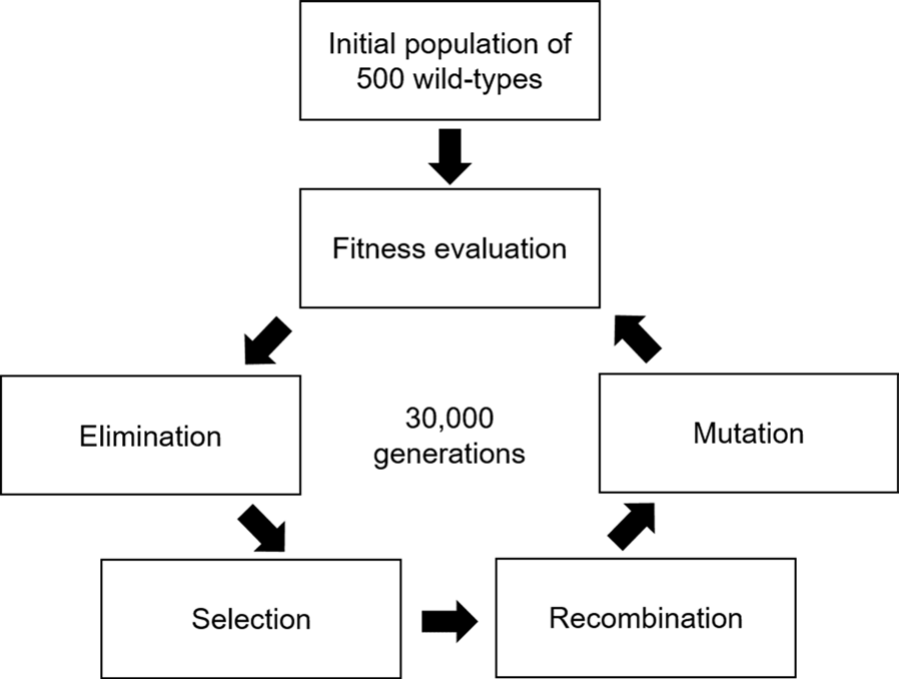
Schematic of genetic algorithm used for in silico evolution of organic acid production

To achieve in silico evolution of organic acid production, a genetic algorithm (GA) was designed. The flux bounds of the reactions in the iDU1756 model were subjected to evolution, with evolutionary pressure to maximise the rate of production of a given organic acid. Each flux bound represented a gene, with the lower bounds being on chromosome 1 and the upper bounds on chromosome 2. The wild-type genes were set as the original flux bounds. The GA was initiated with a population of 500 wild-type individuals, which were subjected to a cycle of fitness evaluation, elimination, selection, recombination, and mutation for a fixed number of generations averaging at 30,000 and ranging from 10,000 to 50,000 depending on the evolutionary goal (Fig. 8).

#### Fitness evaluation

**Fig. 9:**
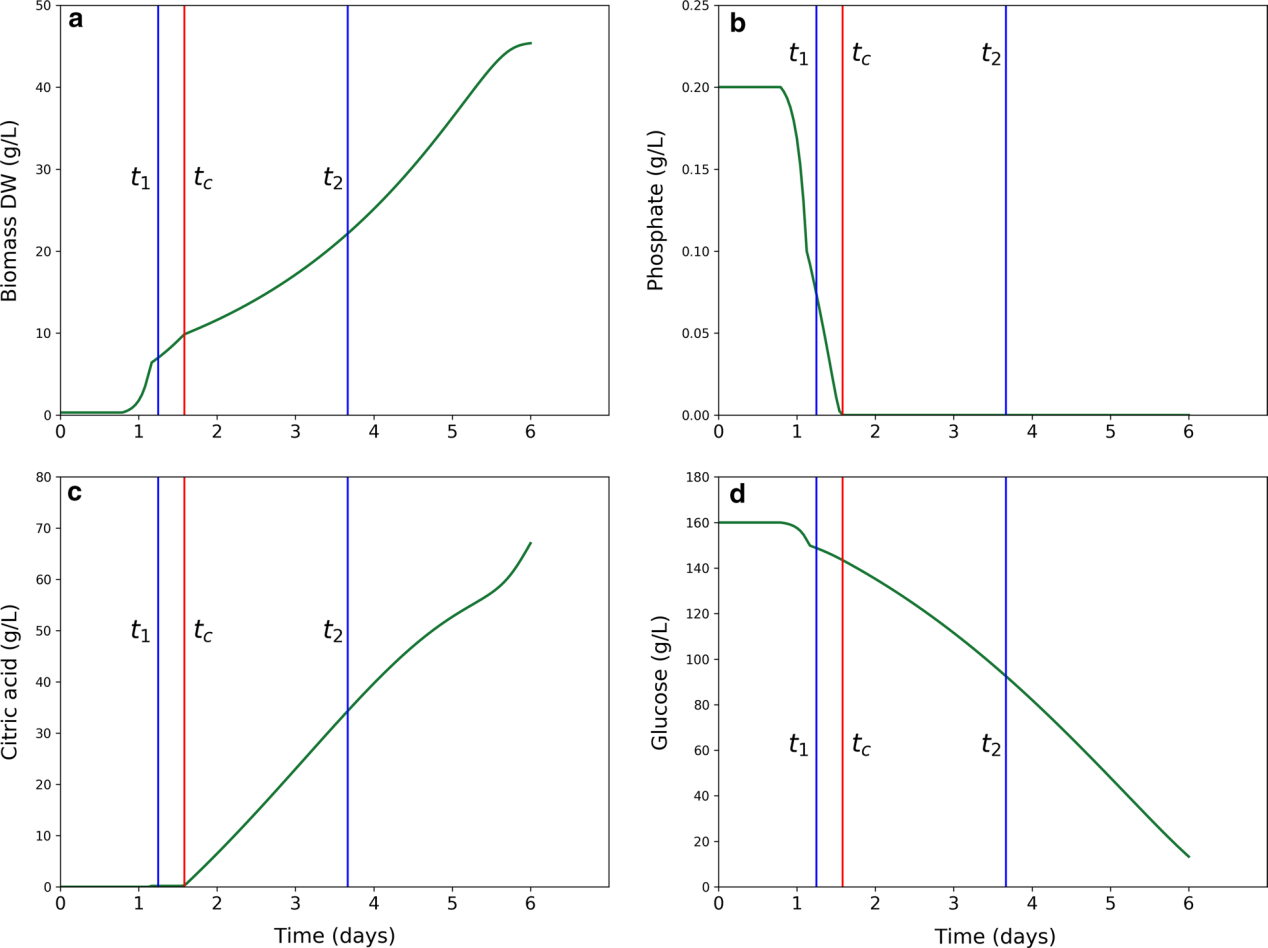
Dynamic modelling of organic acid fermentation as basis of fitness evaluation. The boundary between the two growth phases is shown by the vertical red line, annotated $t_{c}$. The time-points used for fitness evaluation are shown by the vertical blue lines, annotated $t_{1}$ and $t_{2}$

The fitness of new individuals was evaluated by estimating the rate of production of the target acid based on dynamic modelling of organic acid fermentation [8] with the initial pH set to 2, and using the ATCC1015-specific iDU1756 metabolic model. Performing dFBA would evaluate fitness more accurately, however, it is computationally too expensive to be used as the means of fitness evaluation. Therefore, a fitness function was derived that uses flux values from FBA simulations of selected time-points in the two growth phases (Fig. 9), according to

1 $$F = \frac{{c\left( {t_{f} } \right)}}{{t_{f} }},$$

where $F$ is the fitness, $t_{f}$ is the time of substrate depletion, and $c\left( {t_{f} } \right)$ is the target acid yield.

Estimates were calculated for the target acid yield and time of substrate depletion. Growth occurs in two phases; growth phase 1 (phosphate storage), and growth phase 2 (proton production). The boundary is defined by the time $t_{c}$. The fluxes from these growth phases used to calculate estimates are shown in Table 6.

**Table 6:** Fluxes used to calculate estimates of target acid yield and time of substrate depletion for fitness evaluation

Growth phase 1 *t*_0_ < *t* < *t*_c_	$\mu_{1}$	Specific growth rate
	$f_{1}$	Substrate input flux (e.g. glucose)
	$q$	External phosphate input flux
Growth phase 2 *t*_c_ < *t* < *t*_f_	$\mu_{2}$	Specific growth rate
	$f_{2}$	Substrate input flux (e.g. glucose)
	$p_{2}$	Target acid output flux

The fluxes from the time-point in growth phase 1 were used to estimate $t_{c}$, $A_{c}$, and $S_{c}$, where $t_{c}$ is the time of external phosphate depletion and switch to growth phase 2, and $A_{c}$ and $S_{c}$ are the amounts of biomass and substrate at $t_{c}$, respectively

The target acid yield was estimated according to

2 $$c\left( {t_{f} } \right) = \frac{{p_{2} S_{c} }}{{f_{2} }}.$$

The time of substrate depletion was estimated according to

3 $$t_{f} = \left\{ {\begin{array}{ll} {t_{c} + \frac{1}{{\mu_{2} }}ln\left( {1 + \frac{{S_{c} \mu_{2} }}{{f_{2} A_{c} }}} \right), \quad \mu_{2} > 0} \\ {t_{c} + \frac{{S_{c} }}{{f_{2} A_{c} }}, \quad \quad \quad \quad \quad \;\mu_{2} = 0} \\ \end{array} } \right.$$

The equations for $t_{c}$, $A_{c}$, and $S_{c}$ are given in Additional file 7, as well as information on how these and the above equations were derived.

A simplification was made by only applying the mutant flux bounds to growth phase 2, and using wild-type flux bounds in growth phase 1. This assumed that mutations become active at time $t_{c}$, and are switched off during growth phase 1. This simplification was necessary to the performance of the GA, as mutations that affect growth have conflicting effects on fitness when applied to both growth phases. An FBA simulation of a time-point in growth phase 1 was no longer required, which improved the computational efficiency of fitness evaluation. A number of variables became constants by applying wild-type flux bounds to growth phase 1, including $t_{c}$, $A_{c}$, and $S_{c}$. The values for these constants were accurately determined from a dFBA simulation, avoiding the need for estimation using equations given in Additional file 7.

To evolve production of organic acids not produced by the wild-type, adaptations to the fitness function were required. In such cases, the fitness evaluated to zero in wild-type individuals, preventing evolutionary progress. To fix this, fitness was evaluated as the sum of the estimated rates of proton production and target acid production, with different weights given to each. To ensure stronger evolutionary pressure towards production of the target acid over proton production, a higher weight was given to target acid production. The fitness function was adapted to

4 $$F = \frac{{h\left( {t_{f} } \right) + 10c\left( {t_{f} } \right)}}{{t_{f} }},$$

where $h\left( {t_{f} } \right)$ is the proton yield, estimated according to

5 $$h\left( {t_{f} } \right) = \frac{{hS_{c} }}{{f_{2} }},$$

where $h$ is the proton output flux.

The adapted fitness function was applied in evolution of succinic, lactic, malic, acetic, and gluconic production, since none of these organic acids are produced by the wild-type at initial pH 2. The original fitness function was applied in evolution of citric acid production, which occurs in the wild-type.

#### Elimination

Each generation, 5% of the population (25 individuals) were eliminated. The individuals in the population were ranked based on fitness, and the eliminated individuals were randomly drawn from the bottom 50%. Therefore, individuals with fitness in the bottom 50% had an equal chance of elimination, and individuals with fitness in the top 50% were safe from elimination. This enabled individuals with lower fitness to enter the population with some chance of reproduction, before being eliminated. This was found to be beneficial as some individuals with higher fitness have mutations that individually decrease fitness yet increase fitness when combined.

#### Selection

To fill the 5% population gap caused by elimination, new individuals were created. Each new individual was created from two parents, so to fill a 5% gap required a 10% selection (50 individuals). The selection was split across three sources, and did not include any individuals marked for elimination or already selected. 1% (5 individuals) were drawn randomly from across the population. 2% (10 individuals) were drawn from wild-types. The remaining 7% (35 individuals) were drawn randomly from individuals with fitness in the bottom 50%. The percentage from across the population was set such that individuals with fitness in the top 50% reproduced at a sufficiently low rate to prevent their otherwise rapid dominance of the gene pool. This minimised the risk of trapping the evolution on a single solution, allowing distinct solutions to evolve. The percentage from wild-types was set such that a sufficient population of wild-type genes was maintained in the gene pool, allowing the complementation of non-beneficial mutations by recombination. Although the initial population was completely wild-type, the wild-type genes were eventually lost from the gene pool unless re-introduced during the selection stage.

#### Recombination

A new individual was created by recombination of the genes of its two selected parents. Recombination was designed such that most genes were inherited from one parent and a few from the other parent, according to the recombination rate. The recombination rate was set to 0.04, therefore on average 96% of genes were inherited from one parent and 4% from the other parent. The dominant parent was randomly chosen from the two parents.

#### Mutation

Once a new individual was created through recombination of its two parents, its genes were subjected to mutation according to the mutation rate. The mutation rate was set to 0.02, and genes were mutated in a random order. Some genes were protected from mutation, including those corresponding to biomass reactions, the maintenance ATP reaction, and transport reactions. A gene was mutated by adding a small positive or negative value to the flux bound, determined by the Laplace function. Wild-type flux values were used as starting points for mutation in the case of wild-type genes. Mutant genes were mutated from the current mutant flux bound. No flux bounds were allowed to mutate below the original lower bound or above the original upper bound, which prevented irreversible reactions from being made reversible. The location parameter of the Laplace function, $\mu$, was set to zero. The scale parameter of the Laplace function, $b$, was relative to either the wild-type flux or the maximum wild-type flux, or was given the default value of 0.01 if these were zero (see Additional file 6: Figure S1). A mutated gene imposed a flux constraint or forced flux on the corresponding reaction. As mutations forcing flux sometimes resulted in no FBA solution, control steps were added to avoid this. If for a given gene the scale parameter of the Laplace function was set to the default value, the value of mutation was made negative for the lower bound and positive for the upper bound. This prevented mutations from forcing flux in these cases. A secondary mutation rate was added for mutations forcing flux, which was set to 0.3. Therefore, only 30% of mutations forcing flux were allowed. This increased the proportion of mutations constraining flux which would otherwise be in equal ratio to mutations forcing flux. The final control step computed the maximum flux and capped the mutation forcing flux to 1% of the maximum flux. This 1% cap allowed multiple mutations to force flux per individual, without leading to no FBA solution. If a mutation were allowed to force 100% of the maximum flux, it would block other mutations from forcing flux in that individual.

### Data analysis of in silico evolution output for target prediction

Output was generated from in silico evolution by logging new individuals that satisfied certain conditions upon fitness evaluation. If the target acid was not produced by the wild-type, any individual that evolved production of the target acid was recorded. In the case of evolution of citric acid production, individuals with fitness greater than the current highest fitness or with fitness greater than 110% of the wild-type fitness were recorded. The flux bounds of recorded individuals were compared with the wild-type to identify mutations. Mutations with no phenotypic effect were filtered out by only recording mutations if the mutant flux was equal to the mutant flux bound.

Solutions were obtained from the output by selecting recorded individuals with a fitness > 95% of the highest fitness. These solutions were then processed to discard mutations that have a minimal effect. The fitness was re-evaluated upon complementation of a mutation with the wild-type while retaining other mutations. If the fitness decreased by > 5% of the current solution fitness, the mutation was retained, otherwise discarded. This filtered out background from the solutions, leaving only the mutations that contribute significantly to the overall solution fitness. The frequency of each mutation was calculated from the filtered solutions, by counting the occurrences of corresponding reaction indices in the mutations of each solution, and dividing by the number of solutions. The information was used to identify mutation hotspots and inform target prediction.

## Supplementary information

**Additional file 1.** The file provides the Additional Tables S1–S14.

**Additional file 2.** The iDU1756 model presented as a spreadsheet.

**Additional file 3.** The iDU1756 model in SBML format.

**Additional file 4.** A detailed complete comparison of the models iDU1756 and iJB1325.

**Additional file 5: Table S15.** Index numbers of mutations and corresponding reactions and mutation effects.

**Additional file 6.** The file provides the additional Figures S1–S15.

**Additional file 7.** Full derivation of the fitness function used for in silico evolution of organic acid production.

## Data Availability

The datasets used and/or analysed during the current study are available from the corresponding author on reasonable request.
