# Supplementary material for: In silico evolution of Aspergillus niger organic acid production suggests strategies for switching acid output
Source: Biotechnol Biofuels. 2020 Feb 24;13:27. doi: 10.1186/s13068-020-01678-z (PMC7038614; doi:10.1186/s13068-020-01678-z)
Supplement: Supplementary file 1 — Additional file 1. The file provides the Additional Tables S1–S14. [file 13068_2020_1678_MOESM1_ESM.docx]

Table S1. iHL1210 reactions for which no hits were found in version 4.0 of the ATCC1015 genome annotation.

| **iHL1210 reaction** | **Description** | **iHL1210 gene assignment** | **Top ATCC1015 hit (v 4.0)** | **e-value** | **identity (%)** | **Top ATCC1015 hit (v 3.0)** | **e-value** | **identity (%)** |
| --- | --- | --- | --- | --- | --- | --- | --- | --- |
| R311 | 2-keto-3-deoxy-L-rhamnonate aldolase | An03g00040 | Aspni7_1145855 | 4e-75 | 44 | ASPNIDRAFT_194527 | 0 | 96 |
| R414 | Phosphoacetylglucosamine mutase | An18g05160 or An18g05170 | No hits |  |  | ASPNIDRAFT_212120 | 2e-153 | 100 |
| R1219 | Glycerol:NADP+ 2-oxidoreductase (glycerone forming) | An04g10070 | Aspni7_1108731 | 1e-92 | 46 | ASPNIDRAFT_183753 | 1e-92 | 46 |
| R1332 | H_2_O transporter | An14g02450 | Aspni7_1161552 | 0 | 84 | ASPNIDRAFT_125829 | 0 | 84 |
| R1419 | D-fructose transport via PEP:Pyr PTS | An11g08360 | No hits |  |  | No hits |  |  |
| R1420 | D-fructose transport via PEP:Pyr PTS | An11g08360 | No hits |  |  | No hits |  |  |
| R1468 | L-arginine transport via ABC system | An13g01100 | No hits |  |  | No hits |  |  |
| R1479 | L-lysine transport via ABC system | An13g01100 | No hits |  |  | No hits |  |  |
| R1483 | L-proline transport via ABC system | An12g06100 | No hits |  |  | No hits |  |  |
| R1493 | Ornithine transport via ABC system | An13g01100 | No hits |  |  | No hits |  |  |
| R1515 | Acetoacetate transport via proton symport | An08g11830 | No hits |  |  | No hits |  |  |

Table S2. New transport reactions in iDU1756.

| **iDU1756 reaction** | **Function** |
| --- | --- |
| BALe<==>BAL | Benzaldehyde uptake from extracellular amygdalin degradation |
| CPGIII-->CPGIIIm | Coproporphyrinogen III transport to mitochondria for heme biosynthesis |
| H2O2<==>H2O2m | Hydrogen peroxide mitochondrial transport, required in heme biosynthesis pathway |
| HCNe<==>HCN | Cyanide uptake, required for intracellular cyanide degradation |
| PHNe<==>PHN | Phenanthrene uptake, required for intracellular phenanthrene degradation |
| PHLe<==>PHL | Phenol uptake, required for intracellular phenol degradation |
| TSTe<==>TST | Testosterone uptake, required for intracellular testosterone biotransformation |
| ANDe<==>AND | Androstenedione export, from intracellular testosterone and dehydroepiandrosterone biotransformation |
| DHANDe<==>DHAND | Dehydroepiandrosterone uptake, required for intracellular dehydroepiandrosterone biotransformation |
| PHANe<==>PHAN | Phenylacetonitrile uptake, required for intracellular phenylacetonitrile degradation |

Table S3. New input/output reactions in iDU1756.

| **iDU1756 reaction** | **Function** |
| --- | --- |
| GALACTANe<==> | Galactan input for galactan degradation |
| D345THBe<==> | Digallate input for digallate degradation |
| IP6e<==> | Phytate input for phytate degradation |
| CLGe<==> | Chlorogenate input for chlorogenate degradation |
| LNMe<==> | Linamarin input for linamarin degradation |
| QCTe<==> | Quercetin input for quercetin degradation |
| AMYDe<==> | Amygdalin input for amygdalin degradation |
| PRNSe<==> | Prunasin input for prunasin degradation |
| MDNe<==> | Mandelonitrile input for mandelonitrile degradation |
| PCNe<==> | Penicillin G input for penicillin G degradation |
| 4NPPe<==> | 4-nitrophenyl phosphate input for 4-nitrophenyl phosphate degradation |
| DIGALURe<==> | Digalacturonate input for digalacturonate degradation |
| 6MSA<==> | 6-methylsalicylate output from 6-methylsalicylate production |
| PHNe<==> | Phenanthrene input for phenanthrene degradation |
| PHLe<==> | Phenol input for phenol degradation |
| TSTe<==> | Testosterone input for steroid biotransformation |
| DHANDe<==> | Dehydroepiandrosterone input for steroid biotransformation |
| PHANe<==> | Phenylacetonitrile input for phenylacetonitrile degradation |
| CAFe<==> | Caffeate output from chlorogenate degradation |
| ACHe<==> | Acetone cyanohydrin output from linamarin degradation |
| FMM<==> | Formamide output from cyanide degradation |
| 6APCNe<==> | 6-Aminopenicillanate output from penicillin G degradation |
| IP2e<==> | 1D-myo-Inositol 1,4-bisphosphate output from phytate degradation |
| 4NPe<==> | 4-nitrophenol output from 4-nitrophenyl phosphate degradation |
| MNNTe<==> | Manninotriose output from stachyose degradation |
| 2PCe<==> | 2-Protocatechoylphloroglucinolcarboxylate output from quercetin degradation |
| COe<==> | Carbon monoxide output from quercetin degradation |
| LDOPA<==> | L-Dopa output from L-Dopa production |
| DHPHA<==> | 3,4-dihydroxyphenylacetaldehyde output from dopamine metabolism |
| DQ<==> | Dopaquinone output from L-Dopa metabolism |
| DMAT<==> | Dimethylallyltryptophan output from tryptophan prenylation |
| MBT<==> | 4-(3-Methylbut-2-enyl)-L-tryptophan output from tryptophan prenylation |
| PHNO<==> | 1-Phenanthrol output from phenanthrene degradation |
| ANDe<==> | Androstenedione output from steroid biotransformation |

Table S4. New compounds in iDU1756.

| **iDU1756 compound** | **KEGG compound** | **Compound name** |
| --- | --- | --- |
| MNNTe | C05404 | Manninotriose (extracellular) |
| GALACTANe | C05796 | Galactan (extracellular) |
| D345THBe | C01572 | Digallate (extracellular) |
| IP6e | C01204 | Phytate (extracellular) |
| IP2e | C01220 | 1D-myo-Inositol 1,4-bisphosphate (extracellular) |
| CLGe | C00852 | Chlorogenate (extracellular) |
| CAFe | C01197 | Caffeate (extracellular) |
| LNMe | C01594 | Linamarin (extracellular) |
| ACHe | C02659 | Acetone cyanohydrin (extracellular) |
| QCTe | C00389 | Quercetin (extracellular) |
| 2PCe | C04524 | 2-Protocatechoylphloroglucinolcarboxylate (extacellular) |
| COe | C00237 | Carbon monoxide (extracellular) |
| AMYDe | C08325 | Amygdalin (extracellular) |
| PRNSe | C00844 | Prunasin (extracellular) |
| MDNe | C00561 | Mandelonitrile (extracellular) |
| HCNe | C01326 | Hydrogen cyanide (extracellular) |
| PCNe | C05551 | Penicillin G (extracellular) |
| 6APCNe | C02954 | 6-Aminopenicillanate (extracellular) |
| 4NPPe | C03360 | 4-nitrophenyl phosphate (extracellular) |
| 4NPe | C00870 | 4-nitrophenol (extracellular) |
| UDPGALF | C03733 | UDP-alpha-D-galactofuranose |
| ALCARm | C02571 | O-Acetylcarnitine (mitochondrial) |
| ALCAR | C02571 | O-Acetylcarnitine |
| LDOPA | C00355 | 3,4-dihydroxy-L-phenylalanine (L-Dopa) |
| DPA | C03758 | Dopamine |
| DHPHA | C04043 | 3,4-dihydroxyphenylacetaldehyde |
| DQ | C00822 | Dopaquinone |
| 6MSA | C02657 | 6-methylsalicylate |
| HCN | C01326 | Hydrogen cyanide |
| FMM | C00488 | Formamide |
| DMAT | C06067 | Dimethylallyltryptophan |
| MBT | C04290 | 4-(3-Methylbut-2-enyl)-L-tryptophan |
| CPGIIIm | C03263 | Coproporphyrinogen III (mitochondrial) |
| PHNe | C11422 | Phenanthrene (extracellular) |
| PHN | C11422 | Phenanthrene |
| PHNO | C11432 | 1-Phenanthrol |
| PHLe | C00146 | Phenol (extracellular) |
| PHL | C00146 | Phenol |
| TSTe | C00535 | Testosterone (extracellular) |
| TST | C00535 | Testosterone |
| ANDe | C00280 | Androstenedione (extracellular) |
| AND | C00280 | Androstenedione |
| DHANDe | C01227 | Dehydroepiandrosterone (extracellular) |
| DHAND | C01227 | Dehydroepiandrosterone |
| PHANe | C16074 | Phenylacetonitrile (extracellular) |
| PHAN | C16074 | Phenylacetonitrile |
| BALe | C00261 | Benzaldehyde (extracellular) |

Table S5. Reaction species corrections made to reactions from iHL1210.

| **iHL1210 reaction** | **iDU1756 reaction** | **KEGG reaction** |
| --- | --- | --- |
| GTP-->D6RP5P+PPI+FOR+H | GTP+3*H2O-->D6RP5P+PPI+FOR+3*H | R00425 |
| CELLUe-->CELLOBe | CELLUe+H2Oe-->CELLOBe | R06200 |
| 3*O2m+2*PPPG9-->2*PPP9m+6*H2Om | 3*O2m+PPPG9m-->PPP9m+3*H2O2m | R03222 |
| SAM+UPGIII-->SAH+PRECOR | 2*SAM+UPGIII-->2*SAH+PRECOR | R03194 |
| Hm+NADPHm+ABUTm<==>NADPm+DMVATm | Hm+NADPHm+ABUTm<==>NADPm+DHMVAm | R05068 |
| DMVATm-->H2Om+OMVALm | DHMVAm-->H2Om+OMVALm | R05070 |
| HICITm+NADm<==>OXAm+CO2m+NADHm | HICITm+NADm<==>OXAm+NADHm | R04862 |
| SAM-->SAH+5MCSN | SAM+CYTS-->SAH+5MCSN | Not found |
| APROP-->ALA+NH3 | APROP+2*H2O-->ALA+NH3 | R03542 |
| ACYBUT-->GLU+NH3 | ACYBUT+2*H2O-->GLU+NH3 | R01887 |
| DCTOL+3*O2+3*NADPH+3*H-->DCDOL+FOR+3*NADP+4*H2O | DCTOL+NADPH-->DCDOL+NADP | R05639 |
| DCDOL+3*NADPH+3*O2-->DCDA+3*NADP | DCDOL+3*NADPH+3*O2-->DCDA+3*NADP+4*H2O | R07509 |
| H2SO3+O2+H2O<==>S+H2O2 | H2SO3+O2+H2O<==>SLF+H2O2 | R00533 |
| ACNL-->INAC+NH3 | ACNL+2*H2O-->INAC+NH3 | R03093 |
| GL+NAD-->GLYN+NADH+H | GL+NADP-->GLYN+NADPH+H | R01039 |

Table S6. Compartmentalisation corrections made to reactions from iHL1210.

| **iHL1210 reaction** | **iDU1756 reaction** |
| --- | --- |
| CPGIII+O2+2*H-->2*CO2+PPPG9+2*H2O | CPGIIIm+O2m+2*Hm-->2*CO2m+PPPG9m+2*H2Om |
| PECTIN+H2O-->METHOL+PECTATE | PECTINe+H2Oe-->METHOLe+PECTATEe |
| PECTATE+H2O-->GALUNT+H | PECTATEe+H2Oe-->GALUNTe+He |
| PECTATE+H2O-->DIGALUR+2*H | PECTATEe+H2Oe-->DIGALURe+2*He |
| DIGALUR+H2O-->2*GALUNT | DIGALURe+H2Oe-->2*GALUNTe |
| FRUCTAN+H2O-->FRU | FRUCTANe+H2Oe-->FRUe |
| XYLAN+H2O-->XYL | XYLANe+H2Oe-->XYLe |
| STAR+H2O-->AMYLS+GLC | STARe+H2Oe-->AMYLSe+GLCe |
| AMYLSe+H2O-->GLC | AMYLSe+H2Oe-->GLCe |
| STAR+H2O-->DEXTRIN | STARe+H2Oe-->DEXTRINe |
| STAR+H2O-->GLC | STARe+H2Oe-->GLCe |
| DEXTRIN+H2O-->GLC | DEXTRINe+H2Oe-->GLCe |
| 13GLUCAN+H2O-->GLC | 13GLUCANe+H2Oe-->GLCe |
| OICAPm+GLU<==>AKG+LEU | OICAP+GLU<==>AKG+LEU |

Table S7. New nitrogen sources in iDU1756.

| **iDU1756 compound** | **KEGG compound** | **Compound name** | **Empirical evidence** |
| --- | --- | --- | --- |
| PHANe | C16074 | Phenylacetonitrile (extracellular) | Hypothetical |

Table S8. New phosphate sources in iDU1756.

| **iDU1756 compound** | **KEGG compound** | **Compound name** | **Empirical evidence** |
| --- | --- | --- | --- |
| IP6e | C01204 | Phytate (extracellular) | da Silva *et al*, 2005 |
| 4NPPe | C03360 | 4-nitrophenyl phosphate (extracellular) | Hypothetical |

Table S9. Biomass composition in iDU1756.

| **Component** | **mmol gDW^-1^** |
| --- | --- |
| ALA | 0.285419 |
| ARG | 0.112059 |
| ASN | 0.063577 |
| ASP | 0.177634 |
| CYS | 0.021777 |
| GLU | 0.280437 |
| GLN | 0.1301 |
| GLY | 0.227706 |
| HIS | 0.055525 |
| ILE | 0.105128 |
| LEU | 0.186022 |
| LYS | 0.217713 |
| MET | 0.027995 |
| PHE | 0.082496 |
| PRO | 0.116563 |
| SER | 0.170194 |
| THR | 0.141109 |
| TRP | 0.039378 |
| TYR | 0.059275 |
| VAL | 0.144508 |
| AMP | 0.0046740733 |
| GMP | 0.0056294467 |
| CMP | 0.0046740733 |
| UMP | 0.0037247467 |
| DAMP | 0.0006457867 |
| DCMP | 0.0006718133 |
| DTMP | 0.0006457867 |
| DGMP | 0.0006718133 |
| GAG | 0.01634 |
| NIG | 0.09652 |
| PSNIG | 0.12806 |
| GGM | 0.14478 |
| 14GLUCAN | 0.14782 |
| 13GLUCAN | 1.03588 |
| CHIT | 0.61674 |
| TAGLY | 0.010083 |
| DAGLY | 0.001009 |
| MAGLY | 0.008912 |
| C140 | 0.000223 |
| C160 | 0.00148 |
| C180 | 0.000245 |
| C181 | 0.001678 |
| C182 | 0.001664 |
| C183 | 0.000047 |
| ERGOST | 0.034062 |
| ERGOSE | 0.010038 |
| MGDG | 0.030053 |
| MGC181 | 0.012374 |
| DGDG | 0.007859 |
| TGDMIPC | 0.000005 |
| CERB1 | 0.00003 |
| CERB2 | 0.000031 |
| GALCER | 0.000025 |
| GLUCER1 | 0.000024 |
| GLUCER2 | 0.000024 |
| CL | 0.001746 |
| PC | 0.005104 |
| PS | 0.0001196667 |
| PE | 0.0116023333 |
| ORN | 0.00553 |
| ICIT | 0.00039 |
| CIT | 0.013 |
| SUCC | 0.00091 |
| FUM | 0.00007 |
| MAL | 0.00065 |
| NAD | 0.0017 |
| NADH | 0.00018 |
| NADP | 0.00014 |
| NADPH | 0.00008 |
| TRE | 0.04 |
| MNT | 0.18 |
| GL | 0.9030928715 |
| EOL | 0.3 |
| AOL | 0.01 |
| BT | 0.0001 |
| COA | 0.0001 |
| FOLATE | 0.0001 |
| HEMEAm | 0.0001 |
| THDP | 0.0001 |
| SIHM | 0.0001 |
| Q | 0.0001 |
| SPMD | 0.0001 |
| PTRC | 0.0001 |
| RGT | 0.0001 |
| DSAM | 0.0001 |
| RIBOFLAVIN | 0.0001 |
| PYDX | 0.0001 |
| CHOR | 0.0001 |
| CYTCCm | 0.0001 |
| ATP | 71.5025735267 |
| H2O | 69.0511151733 |
| ADP | -71.5025795733 |
| PI | -71.5025795733 |

Table S10. Example solution from evolution of citric acid production.

|  |  |  | **Complementation results** | | |
| --- | --- | --- | --- | --- | --- |
| **Index** | **Reaction** | **Mutation effect** | **% Fitness decrease** | **% Acid flux decrease** | **% Growth increase** |
| 707 | DCMP+ATP+H<==>ADP+DCDP | LC | 17.7 | 36.7 | 96.7 |

The mutation effect is given as UC or LC. UC corresponds to a mutation that imposes a flux constraint on the upper bound. LC corresponds to a mutation that imposes a flux constraint on the lower bound. Complementation results are given, showing the effect on fitness, target acid flux, and growth when the mutation is complemented with the wild-type.

Table S11. Example solution from evolution of lactic acid production.

|  |  |  | **Complementation results** | | |
| --- | --- | --- | --- | --- | --- |
| **Index** | **Reaction** | **Mutation effect** | **% Fitness decrease** | **% Acid flux decrease** | **% Growth increase** |
| 759 | ATPm+AMPm+H<==>2*ADPm | LC | 100 | 100 | 56.1 |
| 53 | ACCOAp+H2Op+GLXp-->MALp+COAp+Hp | UC | 100 | 100 | 12.9 |
| 451 | ADPm+PIm+4.5454*Ho-->ATPm+H2Om+4.5454*Hm | UC | 99.8 | 100 | 113.5 |
| 524 | ALA+GLX<==>PYR+GLY | UC | 68.4 | 70.6 | 22.2 |

The example solution is chosen as the best representative of the average solution and based on fitness. The mutation effect is given as UC or LC. UC corresponds to a mutation that imposes a flux constraint on the upper bound. LC corresponds to a mutation that imposes a flux constraint on the lower bound. Complementation results are given for each mutation, showing the effect on fitness, target acid flux, and growth when the mutation is complemented with the wild-type while retaining the other mutations.

Table S12. Example solution from evolution of malic acid production.

|  |  |  | **Complementation results** | | |
| --- | --- | --- | --- | --- | --- |
| **Index** | **Reaction** | **Mutation effect** | **% Fitness decrease** | **% Acid flux decrease** | **% Growth increase** |
| 34 | ACCOAm+H2Om+OAm<==>CITm+COAm+Hm | UC | 99.7 | 100 | 0 |
| 138 | FTHF+H2O-->FOR+THF+H | UC | 70.5 | 70.7 | 0 |
| 26 | D6PGC+NADP-->RL5P+CO2+NADPH | UC | 62.2 | 62.4 | 0 |
| 636 | SAICAR<==>FUM+AICAR | UC | 27.9 | 41.1 | 66.7 |

The example solution is chosen as the best representative of the average solution and based on fitness. The mutation effect is given as UC or LC. UC corresponds to a mutation that imposes a flux constraint on the upper bound. LC corresponds to a mutation that imposes a flux constraint on the lower bound. Complementation results are given for each mutation, showing the effect on fitness, target acid flux, and growth when the mutation is complemented with the wild-type while retaining the other mutations.

Table S13. Example solution from evolution of acetic acid production.

|  |  |  | **Complementation results** | | |
| --- | --- | --- | --- | --- | --- |
| **Index** | **Reaction** | **Mutation effect** | **% Fitness decrease** | **% Acid flux decrease** | **% Growth increase** |
| 458 | ADP+PI+ATPm+H2Om-->ADPm+PIm+ATP+H2O | UC | 99.9 | 100 | 97.4 |
| 36 | ACOm+H2Om<==>ICITm | UC | 99.9 | 100 | 8.8 |

The example solution is chosen as the best representative of the average solution and based on fitness. The mutation effect is given as UC or LC. UC corresponds to a mutation that imposes a flux constraint on the upper bound. LC corresponds to a mutation that imposes a flux constraint on the lower bound. Complementation results are given for each mutation, showing the effect on fitness, target acid flux, and growth when the mutation is complemented with the wild-type while retaining the other mutations.

Table S14. Example solution from evolution of gluconic acid production.

|  |  |  | **Complementation results** | | |
| --- | --- | --- | --- | --- | --- |
| **Index** | **Reaction** | **Mutation effect** | **% Fitness decrease** | **% Acid flux decrease** | **% Growth increase** |
| 28 | R5P<==>RL5P | LC | 99.6 | 100 | 117.2 |
| 268 | KDDGC<==>PYR+GLYAL | UC | 99.6 | 100 | 117.2 |
| 27 | RL5P<==>XUL5P | UC | 99.6 | 100 | 117.2 |
| 9 | BDG6P<==>F6P | UC | 99.6 | 100 | 117.2 |

The example solution is chosen as the best representative of the average solution and based on fitness. The mutation effect is given as UC or LC. UC corresponds to a mutation that imposes a flux constraint on the upper bound. LC corresponds to a mutation that imposes a flux constraint on the lower bound. Complementation results are given for each mutation, showing the effect on fitness, target acid flux, and growth when the mutation is complemented with the wild-type while retaining the other mutations.
