# Supplementary material for: In silico evolution of Aspergillus niger organic acid production suggests strategies for switching acid output
Source: Biotechnol Biofuels. 2020 Feb 24;13:27. doi: 10.1186/s13068-020-01678-z (PMC7038614; doi:10.1186/s13068-020-01678-z)
Supplement: Supplementary file 5 — Additional file 5: Table S15. Index numbers of mutations and corresponding reactions and mutation effects. [file 13068_2020_1678_MOESM5_ESM.docx]

Table S15. Index numbers of mutations and corresponding reactions and mutation effects.

|  |  |  | **Mutation effect** | | | | | |
| --- | --- | --- | --- | --- | --- | --- | --- | --- |
| **Index** | **Reaction** | **Description** | **Succinic** | **Lactic** | **Malic** | **Citric** | **Acetic** | **Gluconic** |
| 6 | ATP+GLC-->ADP+G6P+H | Hexokinase | N | N | N | N | **UC** | N |
| 9 | BDG6P<==>F6P | Glucose-6-phosphate isomerase | N | N | N | N | N | **UC** |
| 10 | G6P<==>ADG6P | Glucose-6-phosphate isomerase | N | N | N | N | **LC** | N |
| 11 | ADG6P<==>BDG6P | Glucose-6-phosphate 1-epimerase | **LC** | N | N | N | N | N |
| 12 | ATP+F6P-->ADP+FDP+H | 6-phosphofructo-1-kinase | N | **UC** | **UC** | N | **UC** | N |
| 14 | FDP<==>T3P2+T3P1 | Fructose-bisphosphate aldolase | N | **UC** | **UC** | N | N | **UC** |
| 15 | T3P2<==>T3P1 | Triosephosphate isomerase | N | **UC** | N | N | **UC** | N |
| 16 | T3P1+PI+NAD<==>13PDG+NADH+H | Glyceraldehyde 3-phosphate dehydrogenase | N | **UC** | N | N | N | **UC** |
| 17 | ADP+13PDG<==>ATP+3PG | Phosphoglycerate kinase | **UC** | **UC** | N | N | N | N |
| 18 | 3PG<==>2PG | Phosphoglycerate mutase | N | **UC** | N | N | **UC** | N |
| 19 | 2PG<==>PEP+H2O | Phosphopyruvate hydratase | **UC** | **UC** | N | N | N | N |
| 20 | ADP+PEP+H-->ATP+PYR | Pyruvate kinase | N | **UC** | N | N | **UC** | **UC** |
| 21 | ATP+PYR+H2O+CO2-->ADP+PI+OA+2*H | Pyruvate carboxylase | N | N | **UC** | N | N | N |
| 22 | ATPm+PYRm+H2Om+CO2m-->ADPm+PIm+OAm+2*Hm | Pyruvate carboxylase | **UC** | N | N | N | **UC** | N |
| 24 | G6P+NADP-->D6PGL+NADPH+H | Glucose 6-phosphate 1-dehydrogenase | **UC** | N | **UC** | N | **UC** | N |
| 25 | D6PGL+H2O-->D6PGC+H | 6-Phospho-gluconolactonase | N | N | **UC** | N | **UC** | N |
| 26 | D6PGC+NADP-->RL5P+CO2+NADPH | Phosphogluconate dehydrogenase | **UC** | N | **UC** | N | **UC** | N |
| 27 | RL5P<==>XUL5P | Ribulose-phosphate 3-epimerase | **UC** | **UC** | **UC** | N | **UC** | **UC** |
| 28 | R5P<==>RL5P | Ribose-5-phosphate isomerase | N | N | **LC** | N | **LC** | **LC** |
| 29 | R5P+XUL5P<==>S7P+T3P1 | Transketolase | **UC** | **LC** | N | N | **UC** | **UC** |
| 30 | E4P+XUL5P<==>F6P+T3P1 | Transketolase | N | **UC** | **LC** | N | **LC** | N |
| 31 | S7P+T3P1<==>E4P+F6P | Transaldolase | **UC** | **LC** | **UC** | N | **UC** | **UC** |
| 34 | ACCOAm+H2Om+OAm<==>CITm+COAm+Hm | Citrate synthase | N | N | **UC** | N | N | N |
| 35 | CITm<==>ACOm+H2Om | Aconitate hydratase | N | **UC** | N | N | N | N |
| 36 | ACOm+H2Om<==>ICITm | Aconitate hydratase | N | **UC** | N | N | **UC** | N |
| 38 | ICITm+NADm-->AKGm+CO2m+NADHm | Isocitrate dehydrogenase (NAD+) | N | **UC** | N | N | N | N |
| 39 | ICIT+NADP-->AKG+CO2+NADPH | Isocitrate dehydrogenase (NADP+) | N | **UC** | N | N | N | N |
| 40 | AKGm+TDPE1m-->AKGE1m+CO2m | alpha-ketoglutarate dehydrogenase | N | **UC** | N | N | N | N |
| 41 | AKGE1m+LPSE2m-->AKGE2m+TDPE1m | Dihydrolipoamide S-succinyl transferase | N | **UC** | N | N | N | N |
| 42 | AKGE2m+COAm+NADm<==>LPSE2m+SUCCOAm+  NADHm | Dihydrolipoamide dehydrogenase | N | **UC** | N | N | **UC** | **UC** |
| 43 | GDPm+PIm+SUCCOAm<==>GTPm+SUCCm+COAm | Succinate CoA ligase (GDP forming) | **UC** | **UC** | N | N | N | N |
| 45 | SUCCm+Qm<==>FUMm+QH2m | Succinate dehydrogenase (ubiquinone) | **UC** | N | N | N | N | N |
| 46 | FUMm+FADH2m-->SUCCm+FADm+Hm | Succinate dehydrogenase | N | **UC** | N | N | N | N |
| 47 | FUM+FADH2m-->SUCC+FADm+Hm | Succinate dehydrogenase | N | **UC** | N | N | **UC** | N |
| 48 | FUMm+H2Om<==>MALm | Fumarate hydratase | N | N | **UC** | N | **UC** | N |
| 51 | MAL+NAD<==>OA+NADH+H | Malate dehydrogenase | N | N | **LC** | N | N | N |
| 52 | ICITp-->SUCCp+GLXp | Isocitrate lyase | N | **UC** | N | N | **UC** | **UC** |
| 53 | ACCOAp+H2Op+GLXp-->MALp+COAp+Hp | Malate synthase | N | **UC** | N | N | N | **UC** |
| 56 | MAL+NADP-->PYR+CO2+NADPH | Malate dehydrogenase (NADP-specific) | **UC** | N | N | N | N | N |
| 60 | ATPm+CITm+COAm-->ADPm+PIm+ACCOAm+OAm | Mitochondrial ATP:citrate lyase | **UC** | N | N | N | N | N |
| 61 | CITm-->OAm+ACm | Mitochondrial citrate lyase | **UC** | N | N | N | N | N |
| 63 | OA+H2O-->OXAL+AC+H | Oxaloacetate hydrolase | N | **UC** | N | N | N | **UC** |
| 72 | FALD+NAD+H2O<==>FOR+NADH+2*H | Formaldehyde dehydrogenase | N | **LC** | N | N | **LC** | **LC** |
| 73 | FOR+O2+H-->H2O2+CO2 | Formate oxidase | N | N | **UC** | N | N | **UC** |
| 76 | COAm+ADHLIPOm+NADm<==>ACCOAm+LIPOm+  NADHm+Hm | Dihydrolipoamide S-acetyltransferase and lipoamide dehydrogenase | N | N | N | N | N | **UC** |
| 77 | H+PYR-->ACAL+CO2 | Pyruvate decarboxylase | **UC** | N | N | N | N | **UC** |
| 82 | H+MTHGXL+NADPH<==>LACAL+NADP | D-Lactaldehyde dehydrogenase (Methylglyoxal reductase) | N | N | N | N | N | **UC** |
| 84 | LAC+NAD<==>PYR+NADH+H | D-lactate dehydrogenase | **LC** | N | N | N | **LC** | **UC** |
| 85 | RGT+MTHGXL<==>LGT | Lactoylglutathione lyase (glyoxylase I) | N | N | N | N | N | **UC** |
| 86 | LGT+H2O-->LAC+RGT+H | Hydroxyacylglutathione hydrolase (glyoxylase II) | N | N | N | N | N | **UC** |
| 104 | GLUm+Hm-->GABAm+CO2m | Glutamate decarboxylase | **UC** | N | N | N | **UC** | **UC** |
| 105 | GABAm+AKGm-->SUCCSALm+GLUm | 4-Aminobutyrate transaminase | **UC** | N | N | N | **UC** | **UC** |
| 106 | SUCCSALm+NADm+H2Om-->SUCCm+NADHm+2*Hm | Succinate-semialdehyde dehydrogenase (NAD(P)+) | N | N | N | N | **UC** | N |
| 136 | METTHF+NAD-->METHF+NADH | Methylenetetrahydrofolate dehydrogenase (NAD+) | N | **UC** | **UC** | N | N | N |
| 137 | METHF+H2O<==>FTHF+H | Methenyltetrahydrofolate cyclohydrolase | N | **UC** | **UC** | N | **UC** | **UC** |
| 138 | FTHF+H2O-->FOR+THF+H | 5-formyltetrahydrofolate deformylase | **UC** | **UC** | **UC** | N | **UC** | **UC** |
| 139 | METTHF+NADPH+H-->MTHF+NADP | Methylenetetrahydrofolate reductase (NADPH) | N | N | N | **UC** | N | N |
| 155 | T3P1+RL5P+GLN-->PYDX5P+GLU+PI+3*H2O+H | 5'-phosphate synthase pdxT subunit | N | N | **UC** | N | N | N |
| 164 | PD+4HBA-->4HP+PPI | polyisopentenylpyrolinate:4-hydroxybenzoate nonaprenyltransferase | N | N | N | **UC** | N | N |
| 169 | 2PMB+SAM-->2PMMB+SAH+H | ubiquinone biosynthesis methyltransferase | N | N | N | **UC** | N | N |
| 173 | D6RP5P+H2O+H-->A6RP5P+NH3 | 2,5-diamino-6-hydroxy-4-(5-phosphoribosylamino)-pyrimidine 2-aminohydrolase | N | N | **UC** | N | N | N |
| 196 | ACCOA+H2O-->COA+AC+H | Acetyl-CoA hydrolase | **UC** | N | N | N | N | N |
| 200 | ACm+COAm+ATPm-->AMPm+PPIm+ACCOAm+Hm | Acetyl-CoA synthase | **UC** | N | N | N | N | N |
| 201 | ACCOAm+H2Om-->COAm+ACm+Hm | Acetyl-CoA hydrolase | **UC** | N | N | N | N | N |
| 208 | GL3P+FADm-->T3P2+FADH2m | Glycerol 3-phosphate dehydrogenase (FAD dependent) | N | **UC** | N | N | **UC** | N |
| 209 | GLYN+ATP-->T3P2+ADP+H | Glycerone kinase | N | N | N | N | N | **UC** |
| 210 | H+T3P2+NADH-->GL3P+NAD | Glycerol 3-phosphate dehydrogenase (NAD+ dependent) | N | **UC** | N | N | N | N |
| 213 | PYR+NADH+H-->LLAC+NAD | L-Lactate dehydrogenase | **UC** | N | N | N | **UC** | N |
| 214 | LLAC+O2-->AC+CO2+H2O | Lactate 2-monooxygenase | **UC** | N | N | N | **UC** | **UC** |
| 217 | E+NADPH+H-->EOL+NADP | Glycerol dehydrogenase | N | N | **UC** | N | N | **UC** |
| 219 | E4P+H2O-->PI+E | Erythrose 4-phosphate phosphatase | N | N | N | N | N | **UC** |
| 222 | EU+ATP-->EU1P+ADP+H | Erythrulose kinase | N | N | N | N | N | **UC** |
| 237 | XUL5P+FALD<==>T3P1+GLYN | Dihydroxyacetone synthase | N | **UC** | **UC** | N | N | **UC** |
| 240 | RIB+NADPH+H<==>RIBOL+NADP | D-Ribose reductase | **UC** | N | **UC** | N | N | **UC** |
| 244 | RL+NADPH+H-->AOL+NADP | ribulose reductase | **UC** | N | **UC** | N | N | **UC** |
| 260 | BDGLC+NADP-->GLCN15LAC+NADPH+H | beta-D-Glucose:NADP+ 1-oxoreductase | N | N | N | N | **UC** | N |
| 262 | GLCN15LAC+H2O-->GLCNT | Non enzymatic reaction | N | N | **UC** | N | **UC** | N |
| 265 | GLCNT+ATP-->D6PGC+ADP+H | Gluconokinase | **UC** | N | **UC** | N | N | N |
| 267 | GLCNT<==>KDDGC+H2O | Gluconate dehydratase | N | N | N | N | N | **UC** |
| 268 | KDDGC<==>PYR+GLYAL | 2-keto-3-deoxygluconate aldolase | N | N | N | N | N | **UC** |
| 274 | G6P<==>G1P | Phosphoglucomutase | N | N | N | **UC** | N | N |
| 288 | MAN6P<==>F6P | Mannose-6-phosphate isomerase | N | N | N | **LC** | N | N |
| 290 | F6P+NADH+H<==>MNT1P+NAD | Mannitol-1-phosphate 5-dehydrogenase | N | N | **UC** | N | **UC** | **UC** |
| 291 | MNT1P+H2O-->MNT+PI | Mannitol-1-phosphatase | N | N | **UC** | N | **UC** | **UC** |
| 292 | MNT+NADP-->FRU+NADPH+H | Mannitol 2-dehydrogenase (NADP+) | N | N | **UC** | N | N | N |
| 305 | ATP+FRU-->ADP+F1P+H | ketohexokinase | N | N | **UC** | N | N | **UC** |
| 306 | F1P<==>T3P2+GLYAL | fructose-bisphosphate aldolase, class II | N | N | **UC** | N | N | N |
| 311 | UDPG+G6P-->UDP+TRE6P | Trehalose-6-phosphate synthase | N | N | N | **UC** | N | N |
| 312 | TRE6P+H2O-->TRE+PI | Trehalose-phosphatase | N | N | N | **UC** | N | N |
| 370 | FKYN+H2O-->FOR+KYN+H | Kynurenine formamidase | N | N | N | N | N | **UC** |
| 371 | KYN+H2O-->ALA+AN+H | Kynureninase | N | N | N | N | N | **UC** |
| 395 | DHSK<==>PCC+H2O | 5-dehydroshikimate dehydrase | N | N | N | N | N | **UC** |
| 407 | UDPG-->UDP+14GLUCAN | 1,4-alpha-Glucan branching enzyme | N | N | **UC** | N | N | N |
| 412 | ACCOA+GA6P<==>COA+NAGA6P+H | Glucosamine-phosphate N-acetyltransferase | **UC** | N | N | N | N | N |
| 413 | NAGA6P+H2O-->GA6P+AC | N-acetylglucosamine-6-phosphate deacetylase | **UC** | N | N | N | N | N |
| 430 | PPP9m-->HEMEBm | protoheme ferro-lyase (protoporphyrin-forming) | N | N | **UC** | N | N | N |
| 433 | HEMEBm-->HEMEOm | heme A farnesyltransferase | N | N | N | **UC** | N | N |
| 444 | QH2m+0.5*O2m-->Qm+H2Om | Ubiquinol oxidase (mitochondrial alternative oxidase) | **UC** | N | N | N | **UC** | N |
| 448 | NADPH+2*FERIm-->NADP+2*FEROm | NADPH-ferrihemoprotein reductase | N | N | N | N | **UC** | N |
| 449 | QH2m+2*FERIm+2*Hm-->Qm+2*FEROm+4*Ho | Ubiquinol-cytochrome c reductase | **UC** | N | N | N | **UC** | N |
| 450 | 2*FEROm+0.5*O2m+6*Hm-->2*FERIm+H2Om+4*Ho | Cytochrome c oxidase subunit I | **UC** | N | N | N | **UC** | N |
| 451 | ADPm+PIm+4.5454*Ho-->ATPm+H2Om+4.5454*Hm | F1F0-ATPase complex | **UC** | **UC** | N | N | N | N |
| 456 | 2*FERIm+LLACm-->PYRm+2*FEROm | Lactic acid dehydrogenase | **UC** | N | N | N | N | N |
| 457 | 2*FERIm+LACm-->PYRm+2*FEROm | Mitochondrial enzyme D-lactate ferricytochrome c oxidoreductase | N | N | N | N | N | **UC** |
| 458 | ADP+PI+ATPm+H2Om-->ADPm+PIm+ATP+H2O | ADP/ATP translocase | **UC** | N | N | N | **UC** | N |
| 463 | 2*H2O2p-->2*H2Op+O2p | Catalase | N | N | **UC** | N | N | **UC** |
| 481 | H+SAM<==>CO2+DSAM | S-adenosylmethionine decarboxylase | N | N | **UC** | N | N | N |
| 491 | NAGLUm+ATPm-->NAGLUPm+ADPm | Acetylglutamate kinase | N | N | **UC** | **UC** | N | N |
| 493 | NAGLUSm+GLUm-->AKGm+NAORNm | Acetylornithine aminotransferase | **UC** | N | N | N | N | N |
| 494 | GLUm+NAORNm-->ORNm+NAGLUm | Glutamate N-acetyltransferase | N | N | N | N | **UC** | N |
| 498 | MTA+HSER+H-->SAM+H2O | S-Adenosyl-L-methionine hydrolase | N | N | **UC** | N | N | N |
| 510 | H2Om+NADPm+GLUGSALm-->GLUm+2*Hm+NADPHm | Delta-1-pyrroline-5-carboxylate dehydrogenase | **UC** | N | N | N | N | N |
| 511 | 2*H2Om+NADm+P5Cm-->GLUm+Hm+NADHm | Delta-1-pyrroline-5-carboxylate dehydrogenase | **UC** | N | N | N | N | N |
| 512 | NAD+3PG<==>H+NADH+PHP | Phosphoglycerate dehydrogenase | N | N | N | N | N | **UC** |
| 513 | PHP+GLU-->AKG+3PSER | Phosphoserine transaminase | N | N | N | N | N | **UC** |
| 514 | H2O+3PSER-->PI+SER | Phosphoserine phosphatase | N | N | N | N | N | **UC** |
| 520 | GLY+THF+NAD-->METTHF+NADH+CO2+NH3 | Aminomethyltransferase | N | **UC** | N | N | N | N |
| 524 | ALA+GLX<==>PYR+GLY | Alanine-glyoxylate transaminase | **UC** | **UC** | N | N | **UC** | **UC** |
| 534 | H2O+PRBAMP-->PRFP | phosphoribosyl-AMP cyclohydrolase | N | N | N | **UC** | N | N |
| 546 | GLUm+OIVALm<==>AKGm+VALm | Branched chain amino acid aminotransferase | N | N | N | **UC** | N | N |
| 569 | ACCOA+HSER<==>COA+OAHSER | homoserine O-acetyltransferase | N | N | N | N | N | **LC** |
| 576 | HCYS+MTHF-->MET+THF | Methionine synthase | N | N | N | **UC** | N | N |
| 580 | H2O+PEP+E4P-->PI+3DDAH7P | 3-deoxy-7-phosphoheptulonate synthase | N | N | N | **UC** | N | N |
| 581 | 3DDAH7P-->PI+DQT | Pentafunctional arom polypeptide | N | N | N | N | N | **UC** |
| 582 | DQT-->H2O+DHSK | 3-dehydroquinase | N | N | N | N | N | **UC** |
| 587 | PEP+SME3P-->PI+3PSME | 3-phosphoshikimate-1-carboxyvinyltransferase | N | N | N | N | N | **UC** |
| 588 | 3PSME-->PI+CHOR | Chorismate synthase | N | N | N | N | N | **UC** |
| 589 | CHOR-->PHEN | Chorismate mutase | N | N | N | **UC** | N | N |
| 598 | PRPP+AN-->H+PPI+NPRAN | Anthranilate phosphoribosyl transferase | N | N | N | N | N | **UC** |
| 599 | NPRAN-->CPAD5P | N-(5'-phosphoribosyl)-anthranilate isomerase | N | N | N | N | N | **UC** |
| 600 | H+CPAD5P-->H2O+CO2+IGP | Indoleglycerol phosphate synthase | N | N | N | N | N | **UC** |
| 606 | 2*H+NADPH+P5C<==>PRO+NADP | Pyrroline-5-carboxylate reductase | N | N | N | N | **UC** | N |
| 608 | NADm+PROm-->2*Hm+NADHm+P5Cm | Proline dehydrogenase | N | N | N | N | **UC** | N |
| 609 | GLUGSALm<==>P5Cm+H2O+H | Non enzymatic reaction | **LC** | N | N | N | N | N |
| 629 | H2O+GLN+PRPP-->GLU+H+PPI+PRAM | Phosphoribosyl-pyrophosphate amidotransferase | N | N | N | **UC** | N | N |
| 632 | H2O+GLN+ATP+FGAR-->GLU+H+PI+ADP+FGAM | 5'-phosphoribosylformyl glycinamidine synthetase | N | N | **UC** | N | N | N |
| 635 | ASP+ATP+CAIR<==>2*H+PI+ADP+SAICAR | Phosphoribosyl amino imidazole-succinocarbozamide synthetase | N | N | **UC** | N | N | N |
| 636 | SAICAR<==>FUM+AICAR | 5'-phosphoribosyl-4-(N-succinocarboxamide)-5-aminoimidazole lyase | N | N | **UC** | N | N | N |
| 694 | ADP+UDP<==>ATP+UMP+H | Cytidylate kinase | N | N | N | **LC** | N | N |
| 696 | UDP+ATP<==>UTP+ADP | Nucleoside-diphosphate kinase | N | N | N | **UC** | N | N |
| 707 | DCMP+ATP+H<==>ADP+DCDP | Cytidylate kinase | N | N | N | **LC** | N | N |
| 759 | ATPm+AMPm+H<==>2*ADPm | Adenylate kinase | N | **LC** | N | N | N | N |
| 760 | GTPm+AMPm+H<==>ADPm+GDPm | Adenylate kinase | N | **UC** | N | N | N | N |
| 787 | CBCCP+ACCOA<==>BCCP+MALCOA | Biotin carboxylase | N | N | N | **UC** | N | N |
| 788 | ATP+BCCP+CO2+H2O<==>ADP+PI+CBCCP+2*H | Biotin carboxylase | N | N | N | **UC** | N | N |
| 789 | MALCOA+ACP<==>MALACP+COA | Malonyl transferase | N | N | **UC** | N | N | N |
| 821 | C140ACP+MALACP-->C16OACP+CO2+ACP | 3-oxoacyl-[acyl-carrier-protein] synthase | N | N | N | N | **UC** | N |
| 825 | 2*H+C140ACP+MALACP+2*NADPH-->C160ACP+ACP+2*NADP+H2O+CO2 | Fatty-acid synthase | N | N | N | N | **UC** | N |
| 882 | H+C170ACP+NADPH+O2<==>C171ACP+NADP+2*H2O | C170-CoA 9-desaturase | N | N | N | **UC** | N | N |
| 1112 | CLCDPDG+PG-->CMP+CL | Cardiolipin synthase | N | N | N | **UC** | N | N |
| 1210 | DAGLYP+H2O-->DAGLY+PI | phosphatidate phosphatase | N | N | N | **UC** | N | N |
| 1211 | 0.024*C120ACP+0.013*C140ACP+0.012*C141ACP+0.002*C150ACP+0.154*C160ACP+0.02*C161ACP+0.008*C162ACP+0.002*C170ACP+0.026*C180ACP+0.374*C181ACP+0.327*C182ACP+0.032*C183ACP+0.006*C200ACP+DAGLY  -->TAGLY+ACP | 1-acylglycerol-3-phosphate acyltransferase | N | N | **UC** | N | N | N |
| 1216 | G+NADH+2*H<==>GLYAL+NAD+H2O | D-Glyceraldehyde:NAD+ oxidoreductase | N | N | N | N | N | **LC** |
| 1221 | ATP+GLYN-->ADP+T3P2+H | ATP:glycerone phosphotransferase | N | N | N | N | N | **UC** |
| 1222 | DAGLY+UDPGAL<==>UDP+MGDG | UDP-galactose:1,2-diacylglycerol 3-beta-D-galactosyltransferase | N | N | **UC** | N | N | N |
| 1237 | HNO3+NADPH+H-->HNO2+NADP+H2O | Nitrate reductase | N | N | N | N | **UC** | N |
| 1239 | HNO3+NADH+H-->HNO2+NAD+H2O | Nitrate reductase | N | N | N | N | **UC** | N |
| 1241 | HNO2+3*NADH+5*H-->NH4OH+3*NAD+H2O | Nitrite reductase | **UC** | N | N | N | **UC** | N |
| 1242 | NH4OH<==>NH3+H2O | Non enzymatic reaction | **UC** | N | N | N | **UC** | N |
| 1596 | ACCOAm+CARm<==>COAm+ALCARm | Mitochondrial acetyl transfer | N | **UC** | N | N | N | N |
| 1598 | ACCOA+CAR<==>COA+ALCAR | Mitochondrial acetyl transfer | N | N | N | N | **LC** | N |

The mutation effect is given as LC, UC or N. LC corresponds to a mutation that imposes a flux constraint on the lower bound. UC

corresponds to a mutation that imposes a flux constraint on the upper bound. N signifies no mutation.
