# Supplementary material for: In silico evolution of Aspergillus niger organic acid production suggests strategies for switching acid output
Source: Biotechnol Biofuels. 2020 Feb 24;13:27. doi: 10.1186/s13068-020-01678-z (PMC7038614; doi:10.1186/s13068-020-01678-z)
Supplement: Supplementary file 6 — Additional file 6. The file provides the additional Figures S1–S15. [file 13068_2020_1678_MOESM6_ESM.docx]

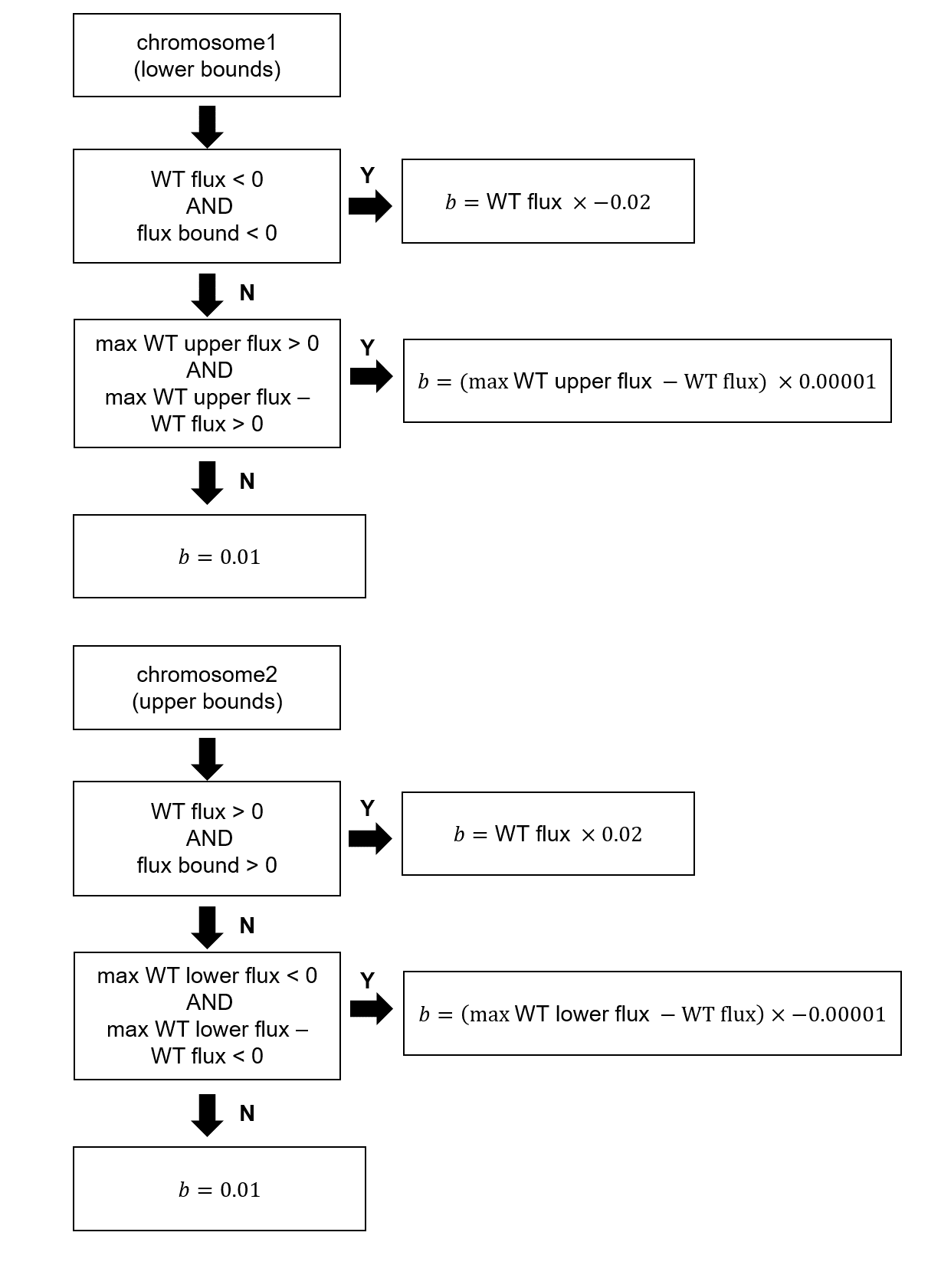
Figure S1. Schematic of procedure used to determine scale parameter of Laplace function.


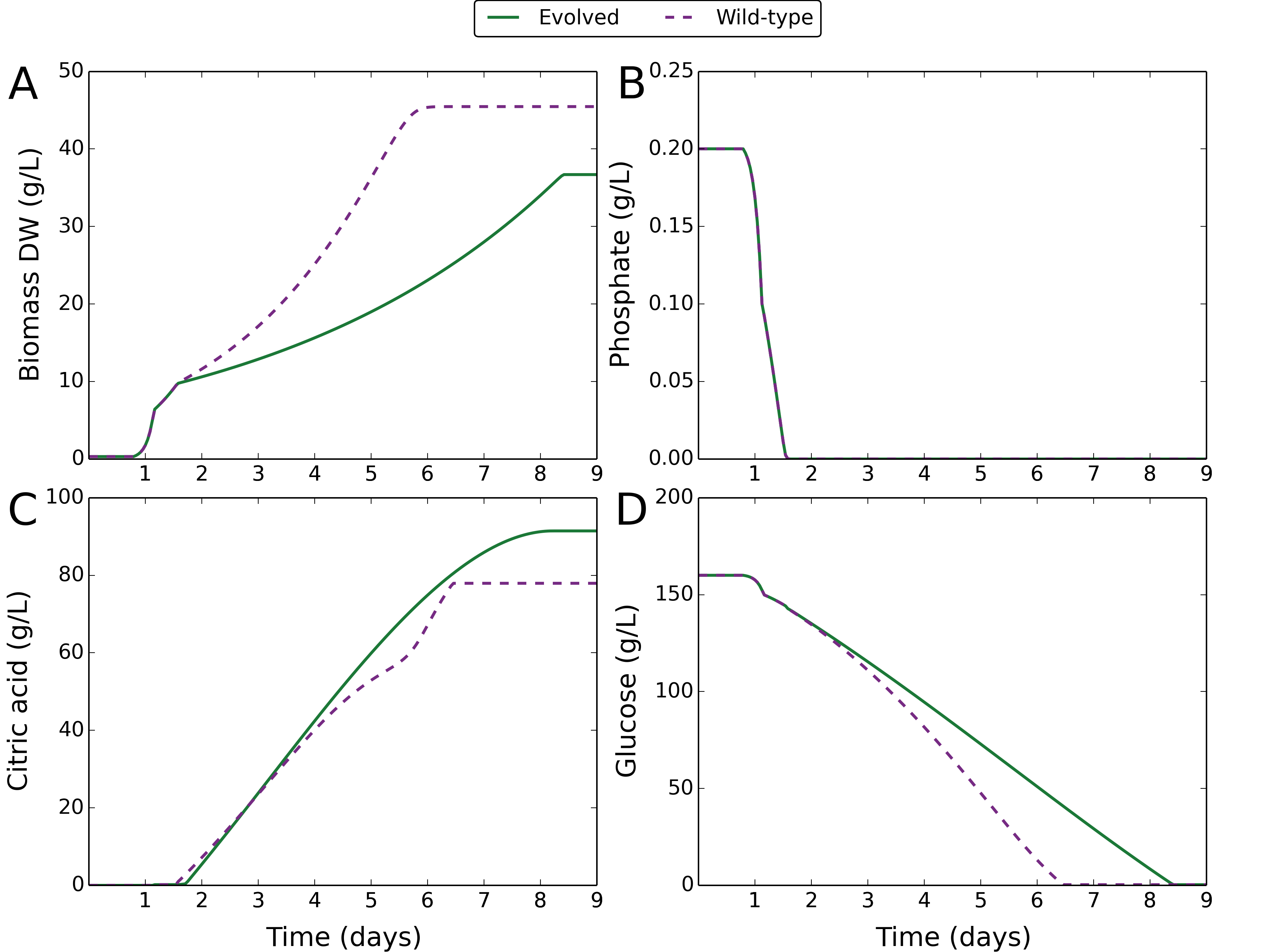


Figure S2. Dynamic modelling of organic acid fermentation comparing the wild-type with a solution from *in silico* evolution towards citric acid production. Green solid lines correspond to an evolved citric acid producer, using a chosen solution (Table S10: see Additional file 1). Purple dashed lines correspond to the wild‑type. Mutations were induced at the point of external phosphate depletion. **(A)** Change in biomass dry weight (g/L) over time. **(B)** Change in external phosphate concentration (g/L) over time. **(C)** Change in external citric acid concentration (g/L) over time. **(D)** Change in external glucose concentration (g/L) over time.


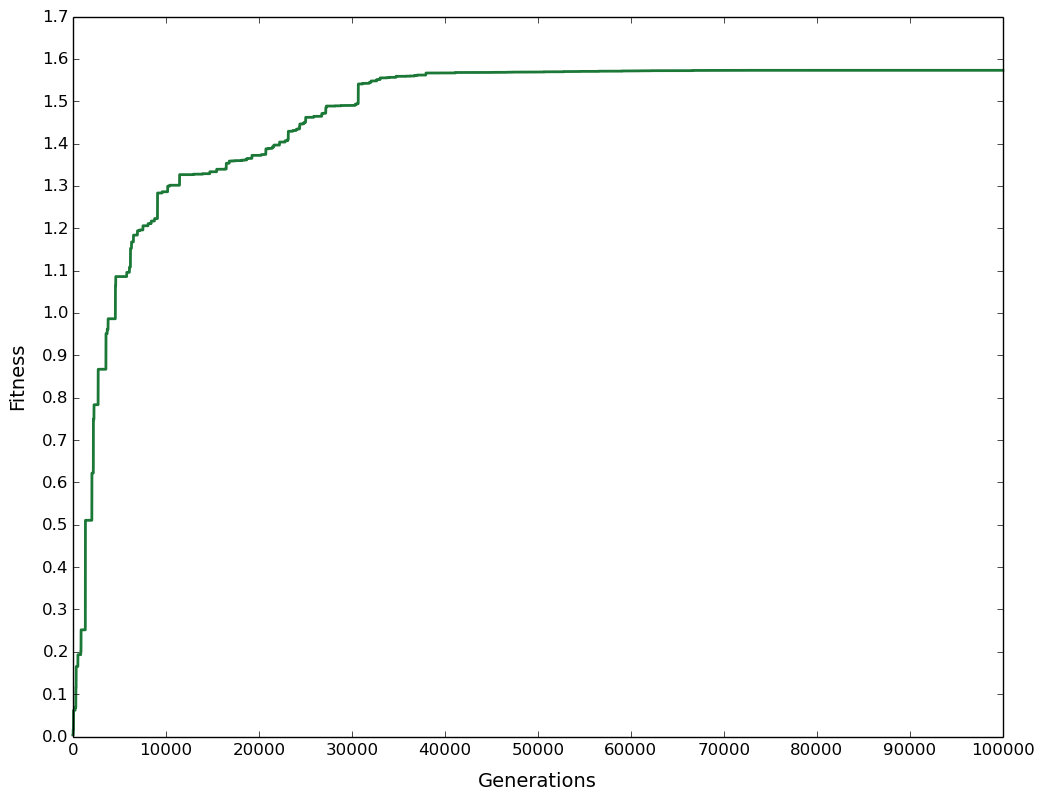


Figure S3. Increase in highest population fitness up to 100,000 generations with evolutionary pressure towards succinic acid production. The evolutionary course of one replicate run is shown.


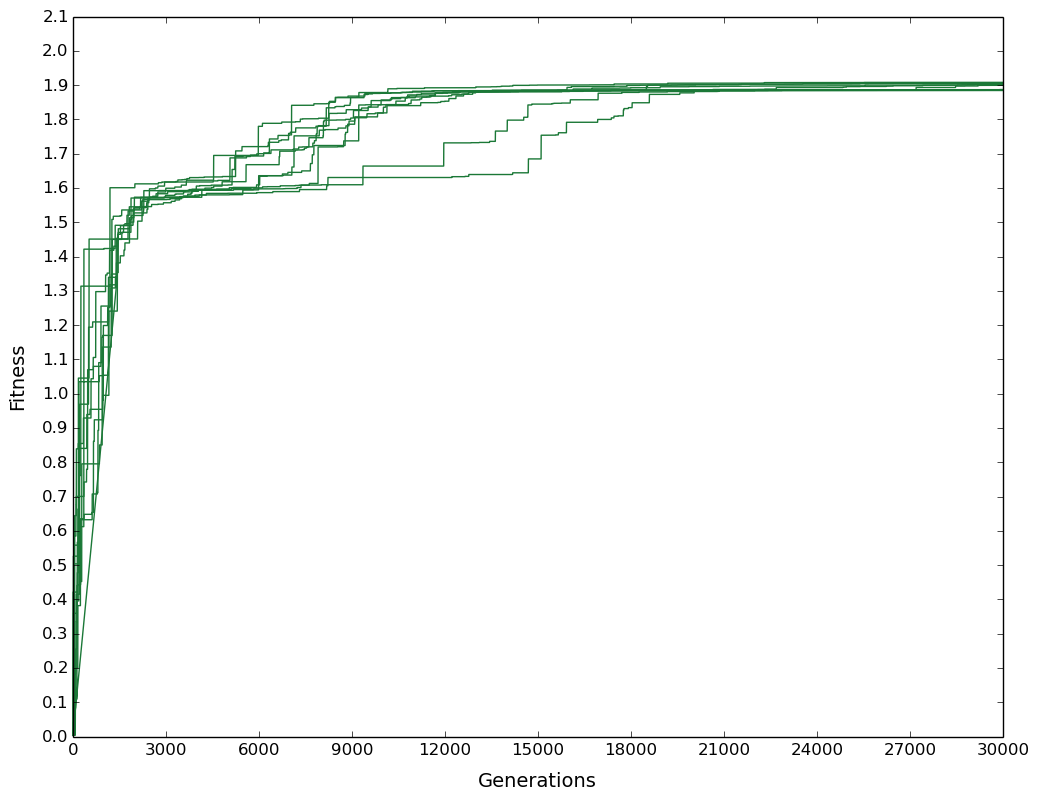


Figure S4. Increase in highest population fitness over generations with evolutionary pressure towards lactic acid production. Each line corresponds to the evolutionary course of one replicate run.


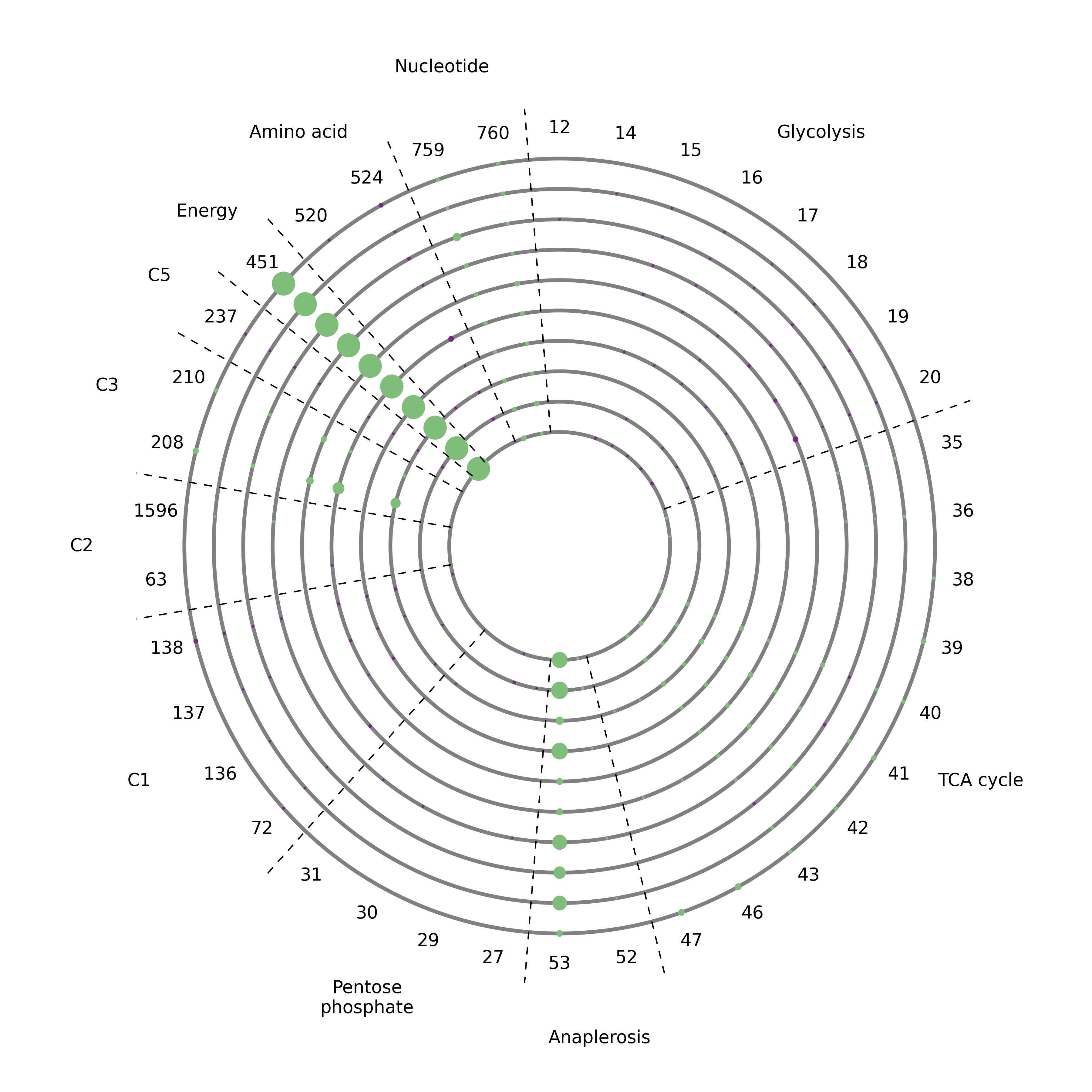

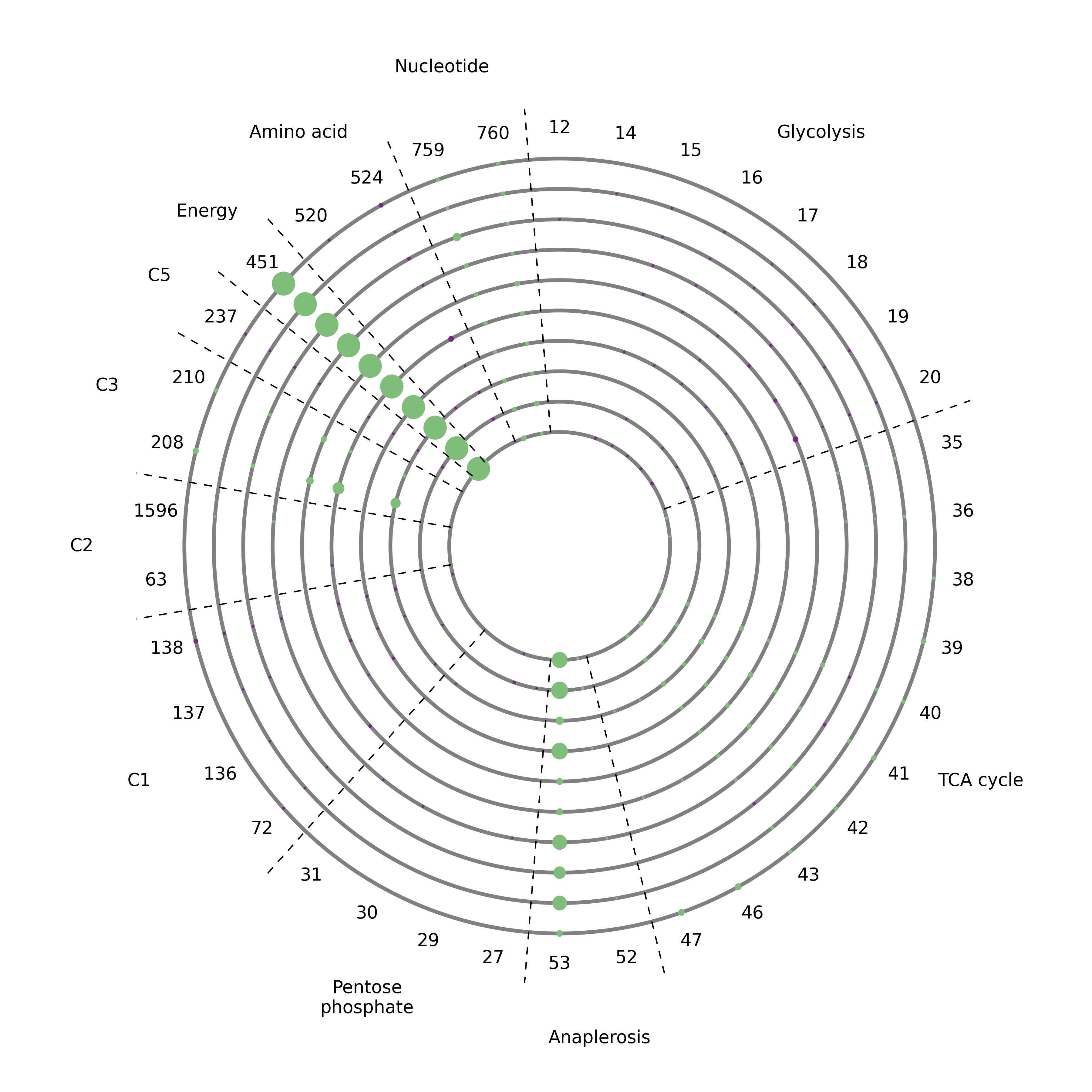


Figure S5. Evolution plot showing the site and frequency of mutations from 10 independent runs with evolutionary pressure towards lactic acid production. Each of the ten grey circles corresponds to the results of one replicate run. The numbers on the outside are indices and refer to reactions where mutations occurred. The corresponding reactions are given in Table S15 [see Additional file 2]. Dots on the grey circles align with these indices and indicate where mutations occurred. The diameter of each dot is proportional to the frequency of the corresponding mutation across solutions from the run. A frequency cut-off of 0.05 was applied. Mutations with a frequency lower than the cut-off are not represented. Green dots indicate mutations that when complemented decrease target acid flux by > 95%. Purple dots indicate mutations that when complemented decrease target acid flux by < 95%. The sectors indicate areas of metabolism that the mutations targeted.


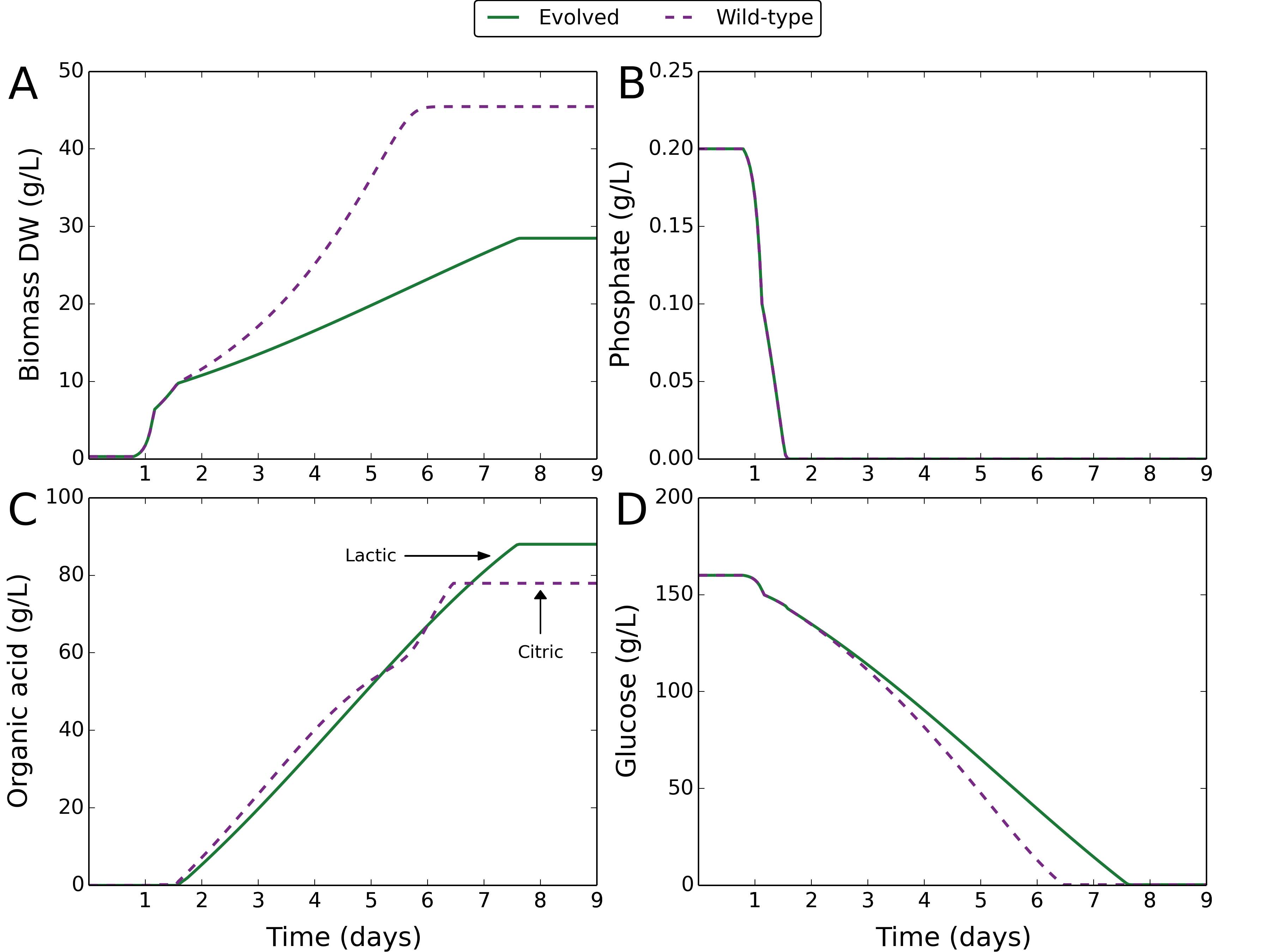


Figure S6. Dynamic modelling of organic acid fermentation comparing the wild-type with a solution from *in silico* evolution towards lactic acid production. Green solid lines correspond to an evolved lactic acid producer, using a solution that best represents the average and based on fitness (Table S11: see Additional file 1). Purple dashed lines correspond to the wild‑type. Mutations were induced at the point of external phosphate depletion. **(A)** Change in biomass dry weight (g/L) over time. **(B)** Change in external phosphate concentration (g/L) over time. **(C)** Change in external organic acid concentration (g/L) over time. Lines are annotated to indicate the organic acid produced. **(D)** Change in external glucose concentration (g/L) over time.


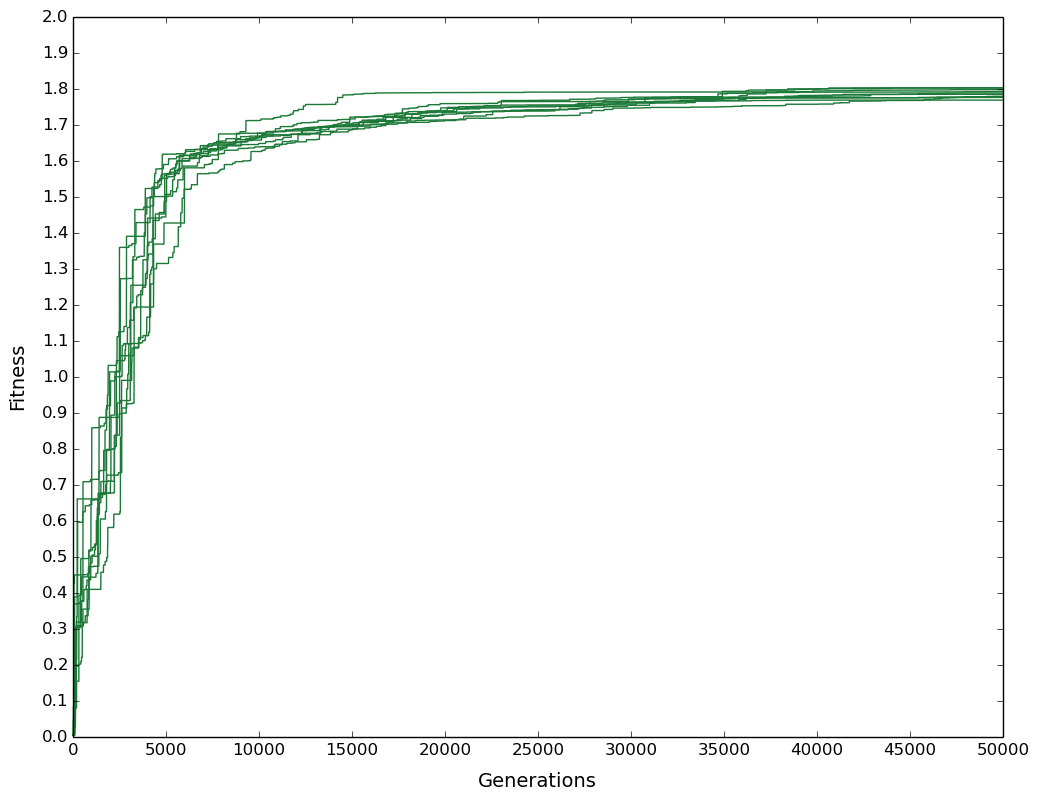


Figure S7. Increase in highest population fitness over generations with evolutionary pressure towards malic acid production. Each line corresponds to the evolutionary course of one replicate run.

Figure S8. Evolution plot showing the site and frequency of mutations from 10 independent runs with evolutionary pressure towards malic acid production. Each of the ten grey circles corresponds to the results of one replicate run. The numbers on the outside are indices and refer to reactions where mutations occurred. The corresponding reactions are given in Table S15 [see Additional file 2]. Dots on the grey circles align with these indices and indicate where mutations occurred. The diameter of each dot is proportional to the frequency of the corresponding mutation across solutions from the run. A frequency cut-off of 0.15 was applied. Mutations with a frequency lower than the cut-off are not represented. Green dots indicate mutations that when complemented decrease target acid flux by > 95%. Purple dots indicate mutations that when complemented decrease target acid flux by < 95%. The sectors indicate areas of metabolism that the mutations targeted
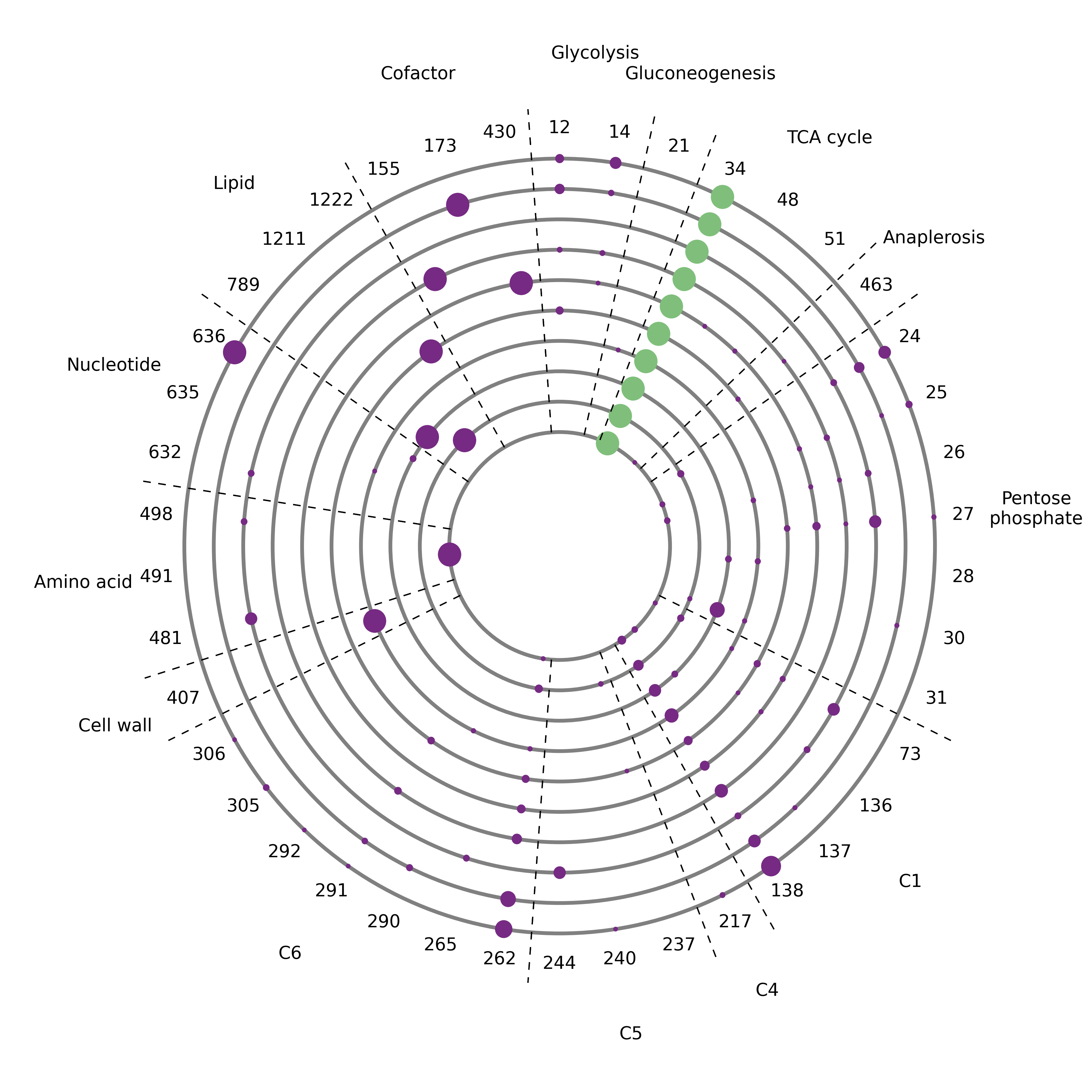
.


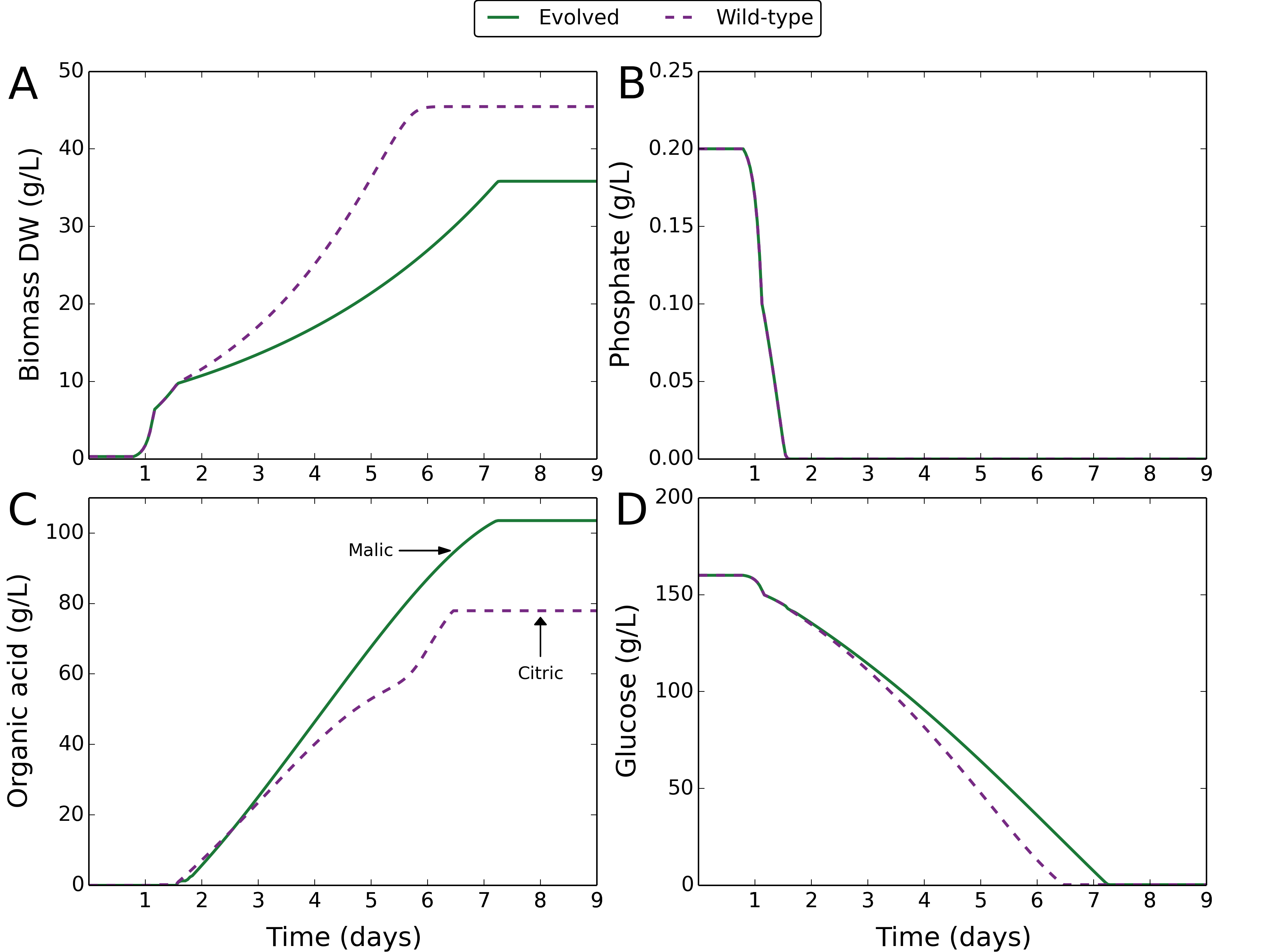


Figure S9. Dynamic modelling of organic acid fermentation comparing the wild-type with a solution from *in silico* evolution towards malic acid production. Green solid lines correspond to an evolved malic acid producer, using a solution that best represents the average and based on fitness (Table S12: see Additional file 1). Purple dashed lines correspond to the wild‑type. Mutations were induced at the point of external phosphate depletion. **(A)** Change in biomass dry weight (g/L) over time. **(B)** Change in external phosphate concentration (g/L) over time. **(C)** Change in external organic acid concentration (g/L) over time. Lines are annotated to indicate the organic acid produced. **(D)** Change in external glucose concentration (g/L) over time.


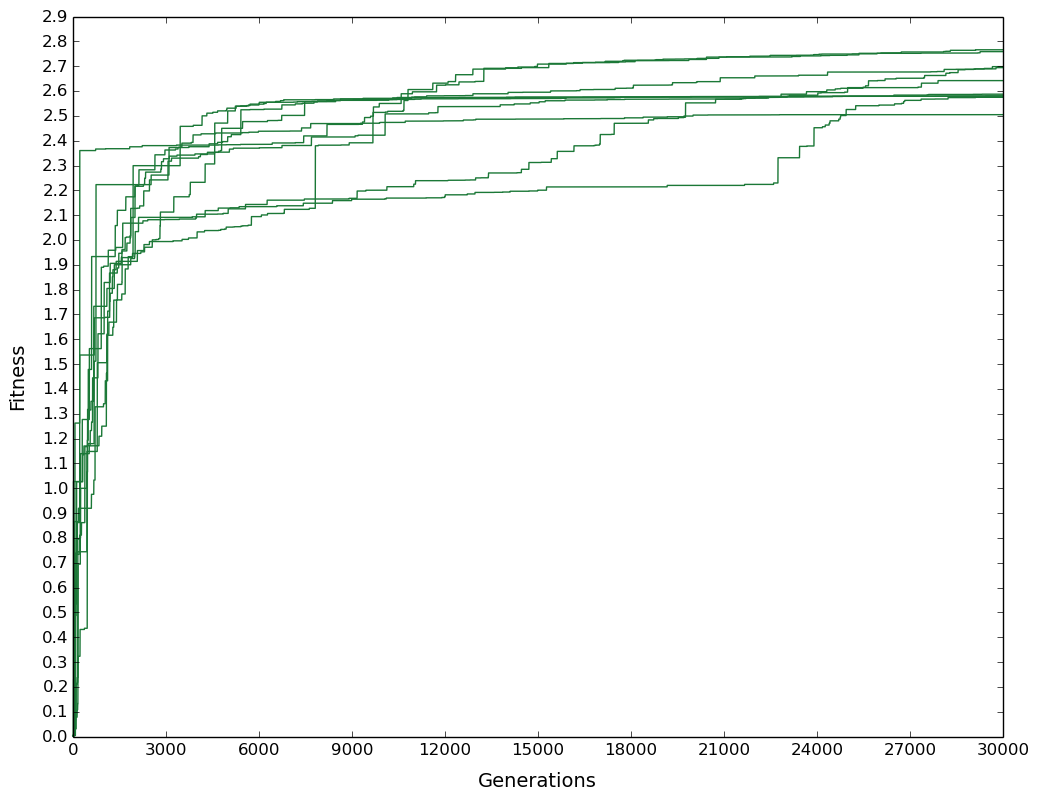


Figure S10. Increase in highest population fitness over generations with evolutionary pressure towards acetic acid production. Each line corresponds to the evolutionary course of one replicate run.


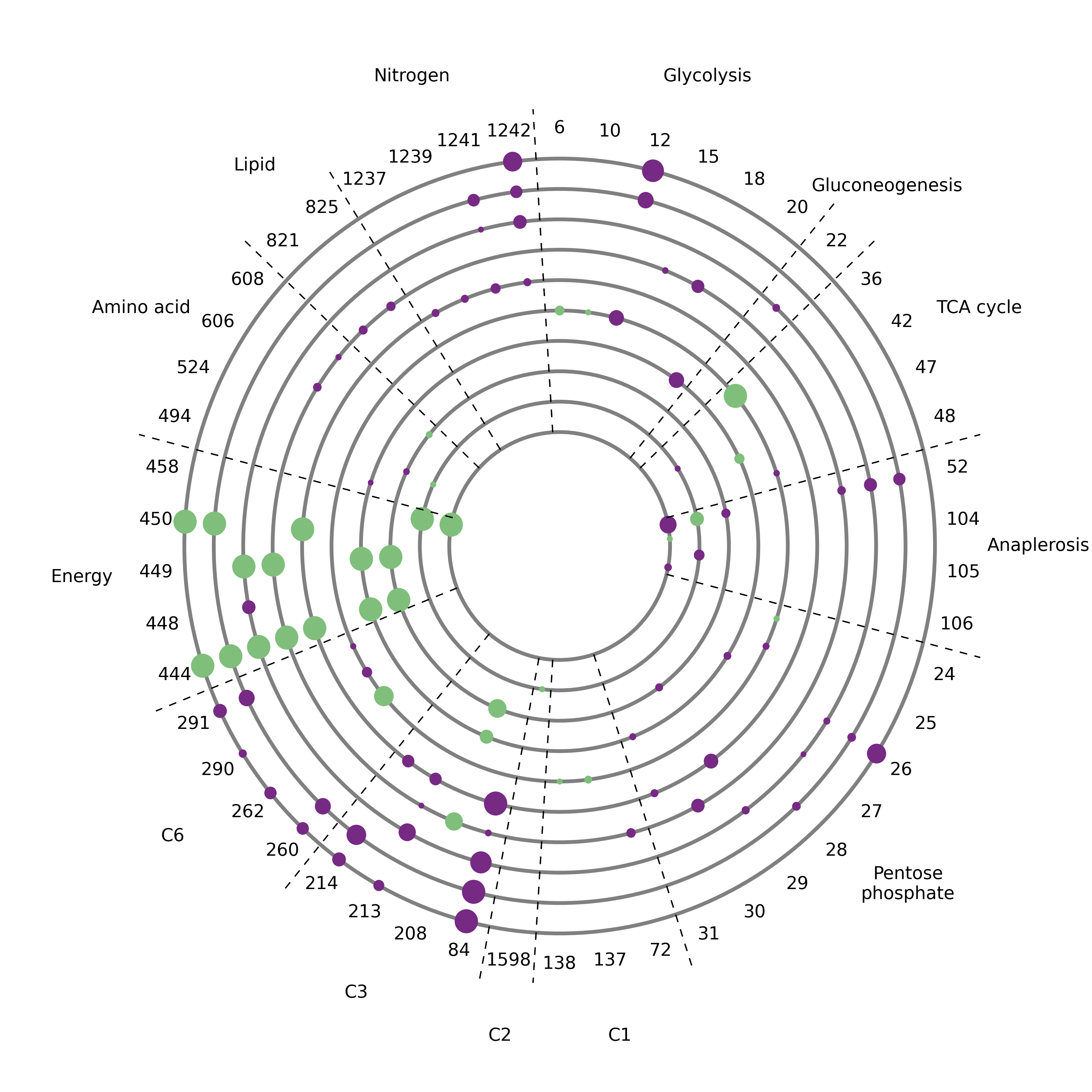

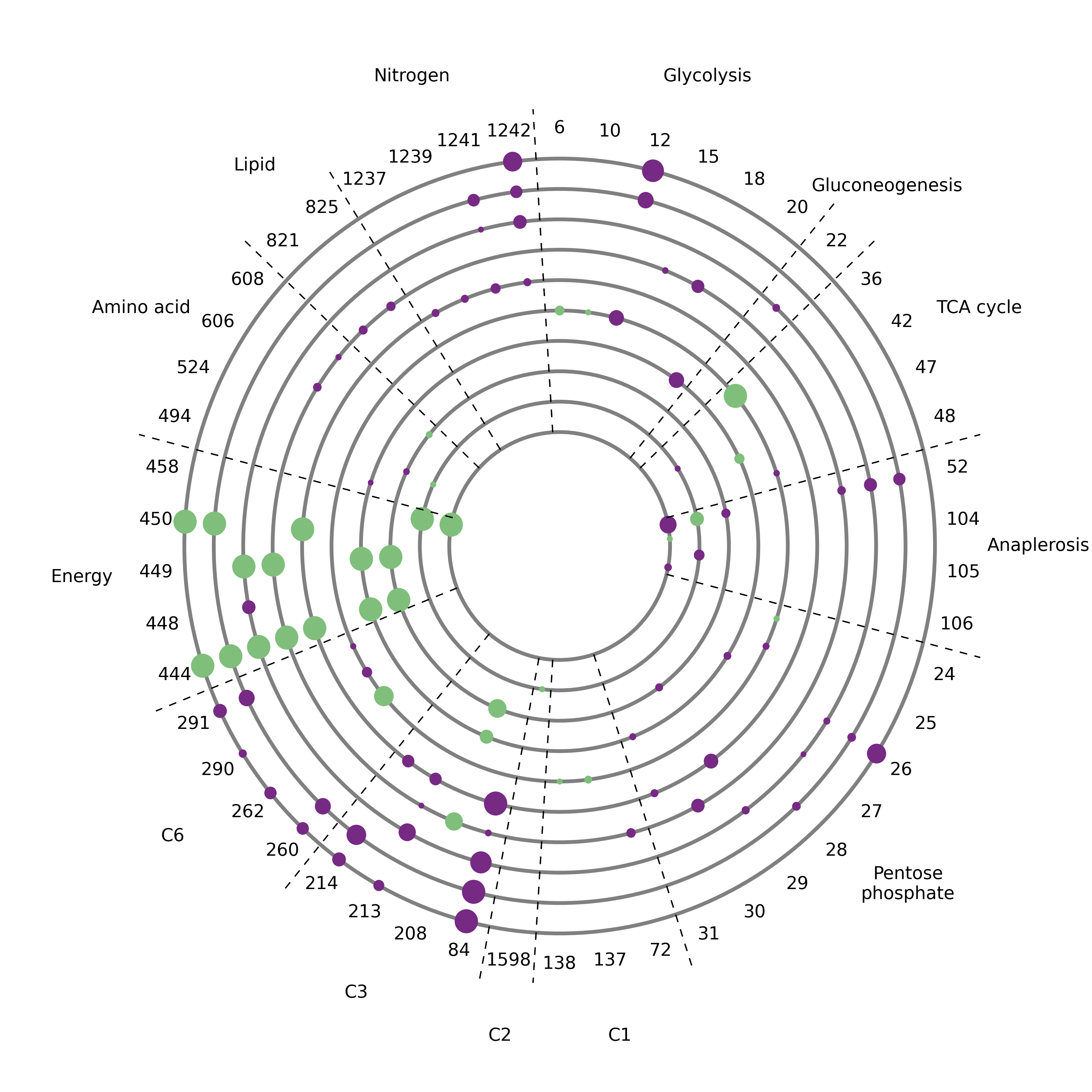


Figure S11. Evolution plot showing the site and frequency of mutations from 10 independent runs with evolutionary pressure towards acetic acid production. Each of the ten grey circles corresponds to the results of one replicate run. The numbers on the outside are indices and refer to reactions where mutations occurred. The corresponding reactions are given in Table S15 [see Additional file 2]. Dots on the grey circles align with these indices and indicate where mutations occurred. The diameter of each dot is proportional to the frequency of the corresponding mutation across solutions from the run. A frequency cut-off of 0.2 was applied. Mutations with a frequency lower than the cut-off are not represented. Green dots indicate mutations that when complemented decrease target acid flux by > 95%. Purple dots indicate mutations that when complemented decrease target acid flux by < 95%. The sectors indicate areas of metabolism that the mutations targeted.


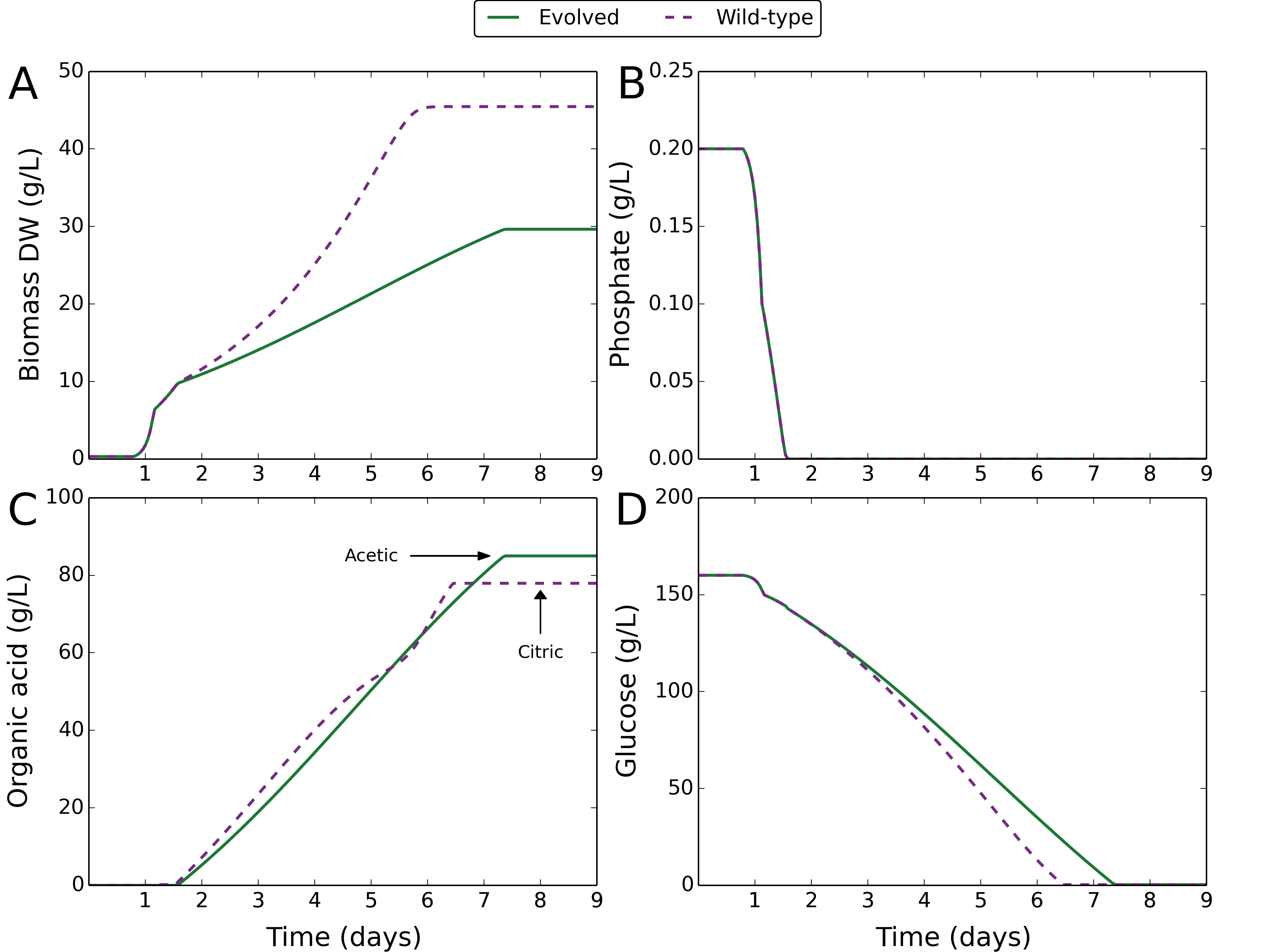


Figure S12. Dynamic modelling of organic acid fermentation comparing the wild-type with a solution from *in silico* evolution towards acetic acid production. Green solid lines correspond to an evolved acetic acid producer, using a solution that best represents the average and based on fitness (Table S13: see Additional file 1). Purple dashed lines correspond to the wild‑type. Mutations were induced at the point of external phosphate depletion. **(A)** Change in biomass dry weight (g/L) over time. **(B)** Change in external phosphate concentration (g/L) over time. **(C)** Change in external organic acid concentration (g/L) over time. Lines are annotated to indicate the organic acid produced. **(D)** Change in external glucose concentration (g/L) over time.


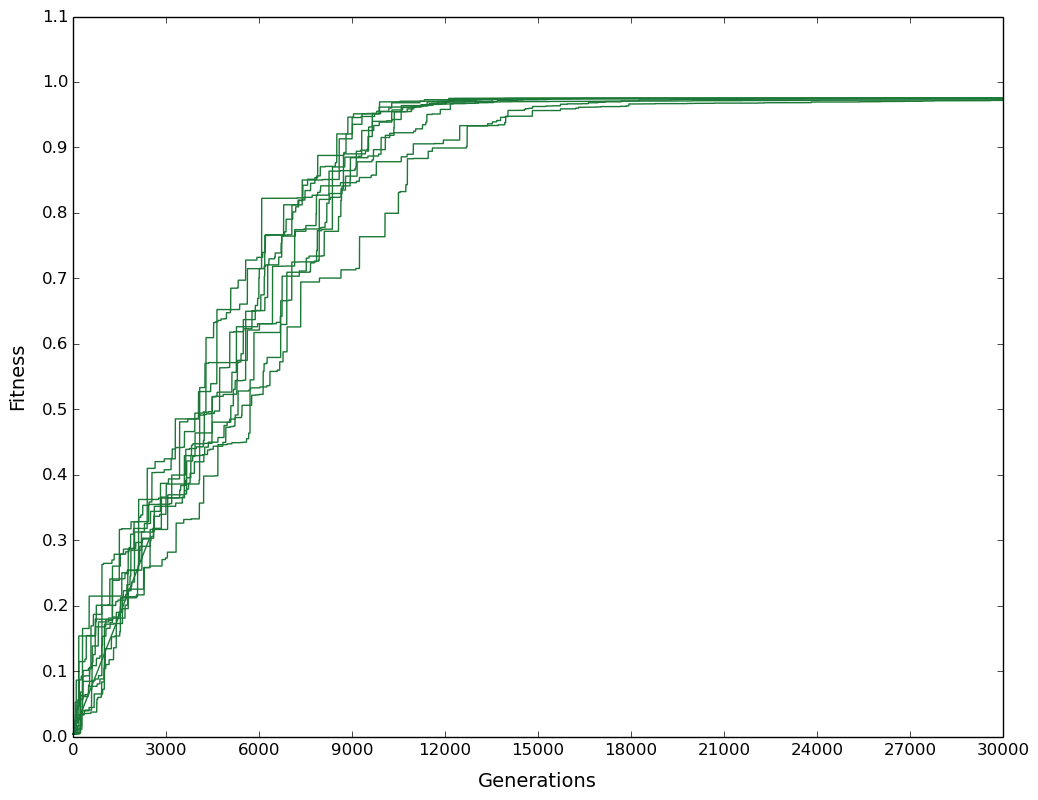


Figure S13. Increase in highest population fitness over generations with evolutionary pressure towards gluconic acid production. Each line corresponds to the evolutionary course of one replicate run.


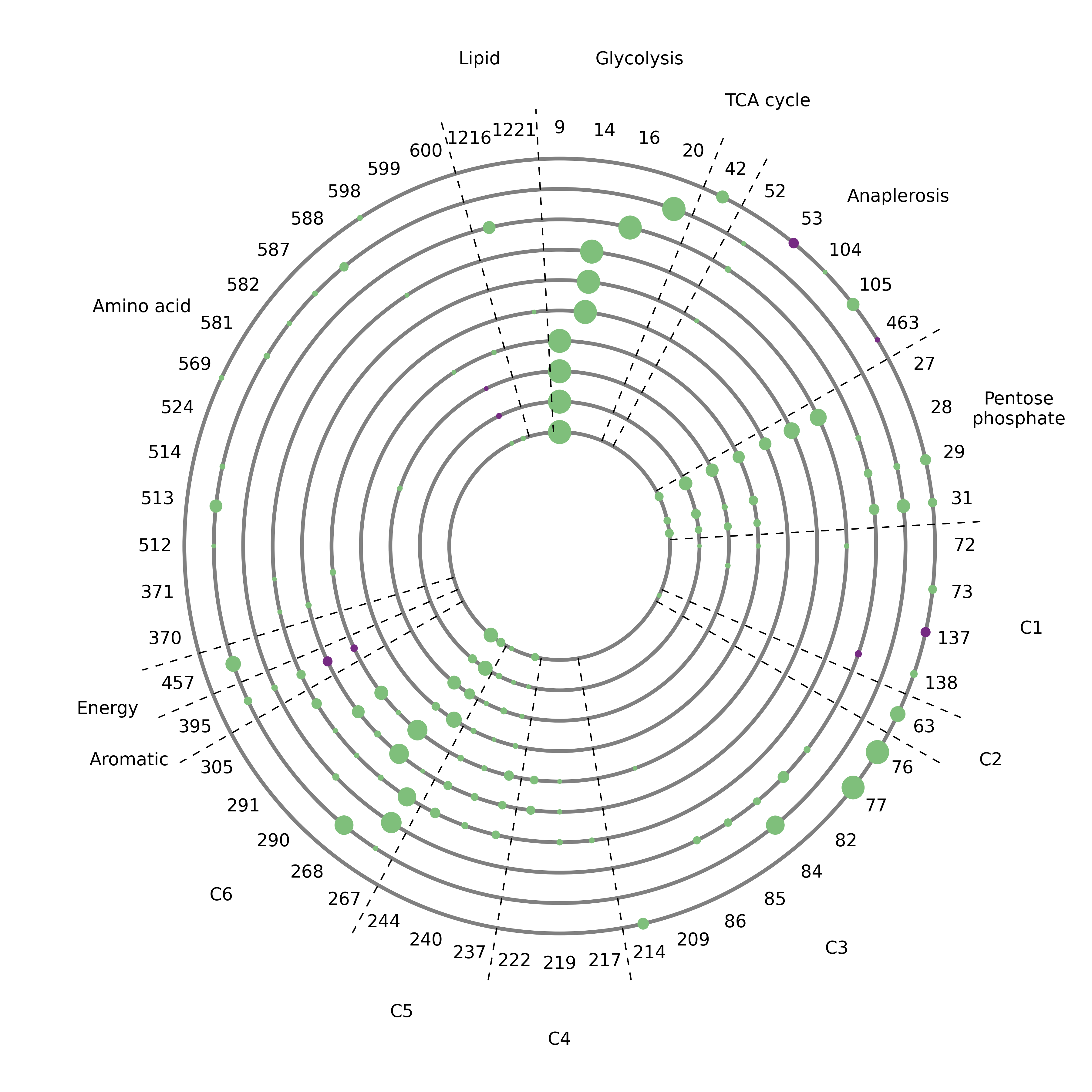
Figure S14. Evolution plot showing the site and frequency of mutations from 10 independent runs with evolutionary pressure towards gluconic acid production. Each of the ten grey circles corresponds to the results of one replicate run. The numbers on the outside are indices and refer to reactions where mutations occurred. The corresponding reactions are given in Table S15 [see Additional file 2]. Dots on the grey circles align with these indices and indicate where mutations occurred. The diameter of each dot is proportional to the frequency of the corresponding mutation across solutions from the run. A frequency cut-off of 0.15 was applied. Mutations with a frequency lower than the cut-off are not represented. Green dots indicate mutations that when complemented decrease target acid flux by > 95%. Purple dots indicate mutations that when complemented decrease target acid flux by < 95%. The sectors indicate areas of metabolism that the mutations targeted.


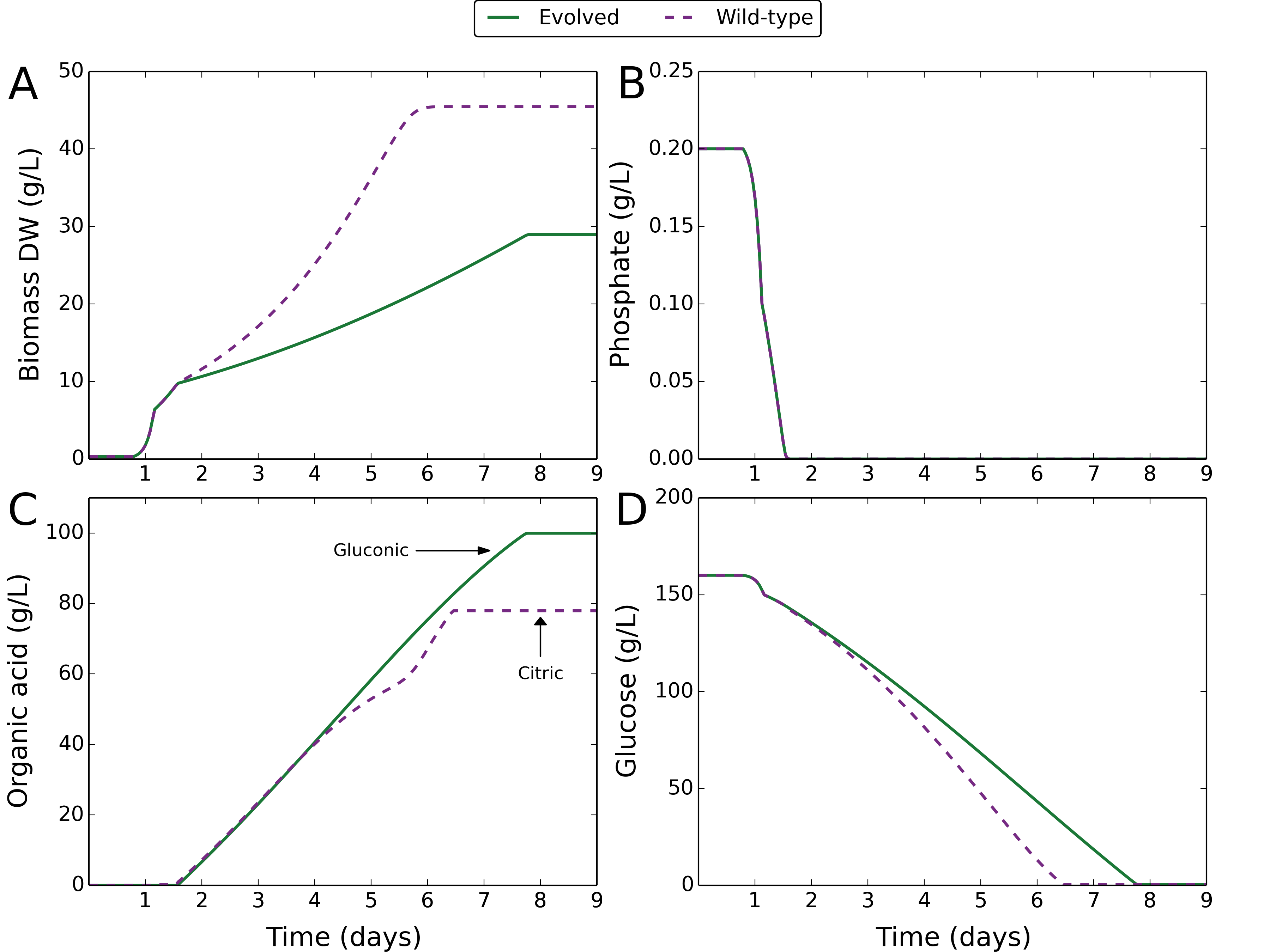


Figure S15. Dynamic modelling of organic acid fermentation comparing the wild-type with a solution from *in silico* evolution towards gluconic acid production. Green solid lines correspond to an evolved gluconic acid producer, using a solution that best represents the average and based on fitness (Table S14: see Additional file 1). Purple dashed lines correspond to the wild‑type. Mutations were induced at the point of external phosphate depletion. **(A)** Change in biomass dry weight (g/L) over time. **(B)** Change in external phosphate concentration (g/L) over time. **(C)** Change in external organic acid concentration (g/L) over time. Lines are annotated to indicate the organic acid produced. **(D)** Change in external glucose concentration (g/L) over time.
