# Supplementary material for: In silico evolution of Aspergillus niger organic acid production suggests strategies for switching acid output
Source: Biotechnol Biofuels. 2020 Feb 24;13:27. doi: 10.1186/s13068-020-01678-z (PMC7038614; doi:10.1186/s13068-020-01678-z)
Supplement: Supplementary file 7 — Additional file 7. Full derivation of the fitness function used for in silico evolution of organic acid production. [file 13068_2020_1678_MOESM7_ESM.docx]

This file gives additional information on the fitness function used in the genetic algorithm for *in silico* evolution of organic acid production. Information is given on how the equations used to estimate target acid yield and time of substrate depletion were derived, as well as equations for $t_{c}$, $A_{c}$, and $S_{c}$, and how these were derived.

The amount of biomass at time $t$, $x\left( t \right)$, was calculated according to

|  | $x\left( t \right)=\left\{ \begin{aligned} \begin{matrix} A_{0}e^{\mu_{1}t}, t_{0}<t<t_{c}, \mu_{1}>0 \\ A_{0},t_{0}<t<t_{c},\mu_{1}=0 \end{matrix} \\ A_{c}e^{\mu_{2}\left( t-t_{c} \right)},t_{c}<t<t_{f},\mu_{2}>0 \\ A_{c},t_{c}<t<t_{f}{, \mu}_{2}=0 \end{aligned} \right.$ | (1) |
| --- | --- | --- |

where $A_{0}$ is the initial biomass at $t_{0}$.

Using the equation for $x\left( t \right)$, the biomass at time $t_{c}$, $A_{c}$, was calculated according to

|  | $A_{c}=\left\{ \begin{aligned} A_{0}e^{\mu_{1}t_{c}},\mu_{1}>0 \\ A_{0},\mu_{1}=0 \end{aligned} \right.$ | (2) |
| --- | --- | --- |

The amount of external phosphate at time $t$, $P_{ex}\left( t \right)$, was calculated according to

|  | $P_{ex}\left( t \right)=\left\{ \begin{aligned} P_{ex}\left( 0 \right)-\frac{qA_{0}}{\mu_{1}}\left( e^{\mu_{1}t}-1 \right),t_{0}<t<t_{c},\mu_{1}>0 \\ P_{ex}\left( 0 \right)-qA_{0}t,t_{0}<t<t_{c},\mu_{1}=0 \end{aligned} \right.$ | (3) |
| --- | --- | --- |

where $P_{ex}\left( 0 \right)$ is the initial external phosphate at $t_{0}$.

The equation for $P_{ex}\left( t \right)$ was derived from the differential equation describing the rate of decrease of external phosphate, which assumed a constant external phosphate input flux

|  | $\frac{dP_{ex}}{dt}=-qx\left( t \right)$ | (4) |
| --- | --- | --- |

Using the equation for $P_{ex}\left( t \right)$, and knowing that external phosphate is zero at time $t_{c}$, an equation for $t_{c}$ was derived

|  | $t_{c}=\left\{ \begin{aligned} \frac{1}{\mu_{1}}ln\left( 1+\frac{\mu_{1}P_{ex}\left( 0 \right)}{qA_{0}} \right),\mu_{1}>0 \\ \frac{P_{ex}\left( 0 \right)}{qA_{0}},\mu_{1}=0 \end{aligned} \right.$ | (5) |
| --- | --- | --- |

The amount of substrate at time $t$, $S\left( t \right)$, was calculated according to

|  | $S\left( t \right)=\left\{ \begin{aligned} \begin{matrix} S_{0}-\frac{f_{1}A_{0}}{\mu_{1}}\left( e^{\mu_{1}t}-1 \right), t_{0}<t<t_{c}, \mu_{1}>0 \\ S_{0}-f_{1}A_{0}t,t_{0}<t<t_{c},\mu_{1}=0 \end{matrix} \\ S_{c}-\frac{f_{2}A_{c}}{\mu_{2}}\left( e^{\mu_{2}\left( t-t_{c} \right)}-1 \right),t_{c}<t<t_{f},\mu_{2}>0 \\ S_{c}-f_{2}A_{c}\left( t-t_{c} \right),t_{c}<t<t_{f}{, \mu}_{2}=0 \end{aligned} \right.$ | (6) |
| --- | --- | --- |

where $S_{0}$ is the initial substrate at $t_{0}$, and $S_{c}$ is the substrate at time $t_{c}$.

The equation for $S\left( t \right)$ was derived from the differential equation describing the rate of decrease of substrate, which assumed a constant substrate input flux

|  | $\frac{dS}{dt}=\left\{ \begin{aligned} -f_{1}x\left( t \right),t_{0}<t<t_{c} \\ -f_{2}x\left( t \right),t_{c}<t<t_{f} \end{aligned} \right.$ | (7) |
| --- | --- | --- |

The equation for $t_{f}$ (time of substrate depletion) was derived from the equation for $S\left( t \right)$, knowing that substrate is zero at time $t_{f}$.

The amount of target acid at time $t$, $c\left( t \right)$, was calculated according to

|  | $c\left( t \right)=\left\{ \begin{aligned} \frac{p_{2}A_{c}}{\mu_{2}}\left( e^{\mu_{2}\left( t-t_{c} \right)}-1 \right),t_{c}<t<t_{f},\mu_{2}>0 \\ p_{2}A_{c}\left( t-t_{c} \right),t_{c}<t<t_{f}, \mu_{2}=0 \end{aligned} \right.$ | (8) |
| --- | --- | --- |

The equation for $c\left( t \right)$ was derived from the differential equation describing the rate of increase of the target acid, which assumed a constant target acid output flux

|  | $\frac{dc}{dt}=p_{2}x\left( t \right)$ | (9) |
| --- | --- | --- |

The equation for $c\left( t_{f} \right)$ (target acid yield) was derived from the equations for $c\left( t \right)$ and $t_{f}$.
